# Acidic open-cage solution containing basic cage-confined nanospaces for multipurpose catalysis

**DOI:** 10.1093/nsr/nwab155

**Published:** 2021-08-20

**Authors:** Kang Li, Kai Wu, Yan-Zhong Fan, Jing Guo, Yu-Lin Lu, Yuan-Fan Wang, Guillaume Maurin, Cheng-Yong Su

**Affiliations:** MOE Laboratory of Bioinorganic and Synthetic Chemistry, Lehn Institute of Functional Materials, School of Chemistry, Sun Yat-sen University, Guangzhou 510275, China; School of Chemistry, South China Normal University, Guangzhou 510006, China; MOE Laboratory of Bioinorganic and Synthetic Chemistry, Lehn Institute of Functional Materials, School of Chemistry, Sun Yat-sen University, Guangzhou 510275, China; MOE Laboratory of Bioinorganic and Synthetic Chemistry, Lehn Institute of Functional Materials, School of Chemistry, Sun Yat-sen University, Guangzhou 510275, China; MOE Laboratory of Bioinorganic and Synthetic Chemistry, Lehn Institute of Functional Materials, School of Chemistry, Sun Yat-sen University, Guangzhou 510275, China; MOE Laboratory of Bioinorganic and Synthetic Chemistry, Lehn Institute of Functional Materials, School of Chemistry, Sun Yat-sen University, Guangzhou 510275, China; MOE Laboratory of Bioinorganic and Synthetic Chemistry, Lehn Institute of Functional Materials, School of Chemistry, Sun Yat-sen University, Guangzhou 510275, China; Institut Charles Gerhardt Montpellier, Centre National de la Recherche Scientifique, École Nationale Supérieure de Chimie de Montpellier, Université de Montpellier, Montpellier 34095, France; MOE Laboratory of Bioinorganic and Synthetic Chemistry, Lehn Institute of Functional Materials, School of Chemistry, Sun Yat-sen University, Guangzhou 510275, China

**Keywords:** open-cage solution, supramolecular cage effect, supramolecular catalysis, cage-confined catalysis, cage-confined nanospaces in solution

## Abstract

The nanoscale chemical spaces inherent in porous organic/coordination cages or solid/liquid materials have been continuously explored for their nanoconfinement effect on selective adsorption and reaction of small gas or organic molecules. Herein, we aim to rationalize the unconventional chemical reactivities motivated by the cage-confined nanospaces in aqueous solutions, where the robust yet permeable nanospaces defined by the open cages facilitate dynamic guest exchange and unusual chemical reactions. The high positive charges on [(Pd/Pt)_6_(RuL_3_)_8_]^28+^ nanocages drive imidazole–proton equilibrium to display a significantly perturbed p*K*_a_ shift, creating cage-defined nanospaces in solution with distinct intrinsic basicity and extrinsic acidity. The supramolecular cage effect plays pivotal roles in elaborating robust solution nanospaces, controlling ingress-and-egress molecular processes through open-cage portals and endowing nanocages with transition-state stabilization, amphoteric reactivities and the phase transfer of insoluble molecules, thus promoting chemical transformations in unconventional ways. Consequently, a wide range of application of cage-confined catalysis with anomalous reactivities may be expected based on this kind of open-cage solution medium, which combines cage nanocavity, solution heterogeneity and liquid-phase fluidity to benefit various potential mass transfer and molecular process options.

## Teaser

Amphoteric and heterogeneous solution nanospaces are created on the basis of permeable open cages with supramolecular cage effect for multirole cage-confined catalysis beyond enzyme mimicking.

## INTRODUCTION

There is a growing interest in exploring and understanding the nanoscale confinement effect imposed in a chemical nanospace because the molecules confined therein can change their physicochemical properties and behaviors as known in nature and biological systems [1,2]. Therefore, the confined chemical nanospaces may offer a promising and exciting area for new chemical and physical research. Such confined nanospaces are not only available from porous organic and metal–organic cages (MOCs) [3–5], but also achievable in porous solids such as zeolites, metal–organic frameworks (MOFs) and covalent organic frameworks (COFs) [6]. Moreover, the unconventional porous liquid MOFs [7], porous liquids based on organic cages or hollow silica spheres [8,9], MOF/cage gels [10–12] and porous ionic/molecular liquids based on varied cages [13,14] are recently discovered. This development trend heralds an interdisciplinary era to utilize plentiful porous materials as synthetic confined systems with the new philosophy of structural design and the counterintuitive state of matter. On the other hand, the combination of the confined nanospaces with the liquid fluidity is deemed to motivate more functionalities with convenient processing techniques, thus intriguing more interests in the exploration of potential applications of the confined nanospaces generable in the solution phase.

**Figure 1.**
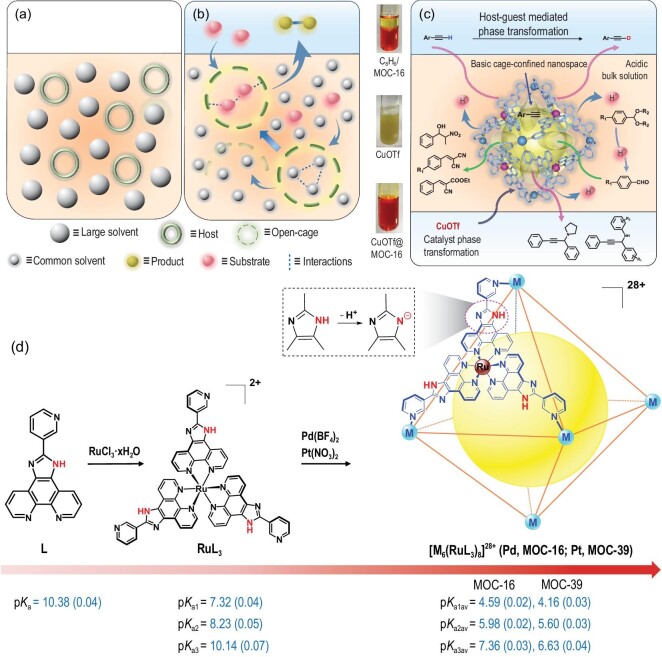
Formation of an open-cage solution as a platform for versatile cage-confined catalysis. (a) Porous liquid designed from rigid hosts dissolved in sterically hindered solvent [15]. (b) Confined solution nanospace created by open cages for the competitive ingress and egress of molecules, where the encapsulation of soluble or insoluble substrates is facilitated by a supramolecular cage effect with preferable intermolecular interactions through replacing solvents or releasing products (see text for discussion). The transient and ill-defined solvent cages are shown for soluble substrates surrounded by solvent molecules. Dashed lines denote the intermolecular interactions. (c) Multiple catalysis mediated by basic cage-confined nanospaces in acidic bulk solution, involving the phase transfer of insoluble substrate, product or catalyst. The tube pictures show the organic/aqueous biphase of phenylacetylene and MOC-16, suspension of CuOTf in the aqueous phase and phase transfer of CuOTf by MOC-16 into a H_2_O–DMSO mixture. (d) Positive-charge-induced p*K*_a_ shifts mediated by imidazole deprotonation.

Typically, a catalytic process by virtue of confined nanospaces demands the dynamic exchange of larger substrates or products with in-and-out priority over smaller solvents that commonly act as reaction media. The open-porous solids have already been widely used in this purpose for selective adsorption/separation and heterogeneous catalysis in routine solvents [6]. The porous liquids, by definition having persistent void space in the liquid state, are obtained from discrete and empty hosts/frameworks in sterically hindered solvents [8,15,16] (Fig. 1a), thereby may not be convenient for the practical application in catalysis when reactants are larger than the solvent molecules. In order to fully utilize the confined nanospaces generable in solution with the nanoconfinement effect for anomalous physicochemical properties and reactivities, a passway between the created solution nanospaces and their application should be opened up, similar to that of open-porous solids, for selective molecular processes and mass transfer. As a consequence, we demonstrate in this work how to take advantage of open-cage solutions, where the robust yet permeable nanospaces are defined by open cages dissolved in solution, to provide an alternative liquid-state basis for multipurpose catalysis with preferential ingress and egress of guest molecules over solvents (Fig. 1b). This target system is reminiscent of natural enzymes, which behave like liquid-phase homogeneous catalysts but with superior performance owing to the buried active sites on the pocket surface of the protein matrix [17,18]. The resulting microenvironment plays a vital role in unique enzyme activity in terms of efficiency and specificity [17,19,20]. In this context, supramolecular open cages containing persistent intrinsic nanocavity and permeable portals are able to mimic the functions of enzymes, featuring solubility in water or organic solvents while being able to merge the distinct attributes of homogeneous, heterogeneous, enzymatic and even phase-transfer catalysis [18–22] into cage-confined catalysis (Fig. 1c).

Different from enzymes that are actually not homogeneously dissolved in aqueous media, the formation of open-cage solutions leads to the generation of the confined nanospaces in homogeneous solution by the dissolved open cages with their permeable walls as boundaries, which function as heterogeneous interfaces for mass transfer, thus imparting solution heterogeneity as in enzymes. The solvent cage effect has long been known to be important for the dissolving and diffusing of solutes [23,24]; nevertheless, the short-lived solvent cages (≈10^−^^11^ s) with ill-defined shapes (Fig. 1b) are inadequate for the generation of robust solution nanospaces [8,15]. Instead, engineering solution nanospaces from open cages is effective if they meet the following prerequisites: (i) possess a rigid shape enclosing a robust intrinsic nanocavity, (ii) have cage portals for selective and dynamic guest exchange and (iii) display solubility and stability in solution. Therefore, the solvent cage effect can be translated into a supramolecular paradigm with the aid of open cages, where the supramolecular cage effect [25] is essential to maintain robust solution nanocavities with properties and functions strikingly different from those of bulk media [26–29] and to control the guest encapsulation priority to preferentially bind substrates over solvents. In a catalytic procedure, the supramolecular cage effect predominates the substrate transportation via selective guest inclusion, making substrates more soluble or even enabling the inclusion of insoluble substrates via phase transfer. This becomes feasible because the process of guest inclusion by open cages is different from the conventional dissolving process of solutes, since work is no longer required to create solvent cages [8]. Moreover, the supramolecular cage effect takes advantage of solvophobicity to facilitate the inclusion of mutual matching substrates, where the entropic loss is counterbalanced by the entropic gain from the release of ordered solvents, and the enthalpic gain by forming favorable host–guest interactions [4] (Fig. 1b). Many types of host–guest interactions contribute to the supramolecular cage effect, including hydrogen bonding, π–π stacking, electrostatic interactions, etc. [30–34], which not only result in a supramolecular confinement effect for unusual reactivity and selectivity [35] but also enable synergistic actions on molecular processes through charge concentration, proton equilibrium and electron/energy transfer that are commonly observed in enzymes. Finally, a catalytic process demands the release of products for reaction turnover number (TON), which is achievable when a host–guest misfit occurs after the chemical transformation of substrates into products [36,37].

## RESULTS AND DISCUSSION

The formation of open-cage solutions is expected to provide a versatile basis for cage-confined catalysis, where the confined solution nanospaces act as reaction vessels because of supramolecular cage effect. Conceptual generalization to combine cage nanospaces with liquid fluidity can open up new avenues for multipurpose catalysis, taking into account the bourgeoning development of supramolecular catalysis with various supramolecular cages [20,34,38–43]. We demonstrate the feasibility of this concept by using a bimetallic [Pd_6_(RuL_3_)_8_]^28+^ nanocage (MOC-16) that is known to feature supramolecular confinement and coupling effects for guest encapsulation and photocatalysis [25,37,44]. Herein, we focus on its unique feature to create basic cage-defined nanospaces in an acidic bulk solution for multipurpose cage-confined catalysis, attributable to its titratable imidazole-NH groups and +28 positive charges (Fig. 1d), in contrast to known negatively charged cages [45,46]. For comparison, a new [Pt_6_(RuL_3_)_8_]^28+^ nanocage (MOC-39) capturing 1,1^′^-binaphthol guests on its open portals is also assembled (Figs S1 and S2; Tables S1 and S2). Both MOCs comprise six Pd/Pt^2+^ cations and eight RuL_3_^2+^ metalloligands that are organized into a truncated octahedron with a 3.2 nm spherical diameter, possessing +28 positive charges, 48 imidazole-N/NH groups and 12 open portals (1.0 nm × 1.4 nm in size). The nanocages are water-soluble with aromatic walls yielding the hydrophobic nanocavities, enabling encapsulation of a variety of guest molecules that are insoluble even in water. We expect that the high charges of the nanocages are able to not only modulate reactivity [47,48], increase the acidity of the encapsulated molecules and facilitate proton abstraction and anionic transition-state stabilization, but also drive proton equilibrium to shift p*K*_a_ thermodynamically via imidazole deprotonation, resembling well-known enzymatic p*K*_a_ perturbation functions [49,50]. Accordingly, intrinsically basic solution nanocavities could be generated in acidic extrinsic media, bringing heterogeneity into aqueous media for multipurpose reactions with the aid of the supramolecular cage effect. Moreover, imidazole-N donors are ready for metal coordination, enabling the phase transfer of insoluble metal catalysts into a solution to separately concentrate on the surface of nanocages, thus turning heterogeneous catalysis into homogeneous catalysis to benefit more catalytic purposes.

The p*K*_a_ values of free L, RuL_3_ metalloligand and MOC-16/39 were quantitatively measured by applying the potentiometric titration method (Fig. 1d; Figs S3–S9 and Tables S3–S9) in aqueous media. Due to the complicated p*K*_a_ values of a nanocage containing 24 imidazole-NH groups, the calculation is simplified according to its high symmetry. Namely, eight RuL_3_ motifs on each octahedral facet are considered to be spatially and chemically equivalent (Fig. S6), thus reducing the calculation to three averaged values of p*K*_a1av_, p*K*_a2av_ and p*K*_a3av_ comparable to p*K*_a1_, p*K*_a2_ and p*K*_a3_ of RuL_3_. Coordination of L with Ru^2+^ causes a decrease in p*K*_a_ from 10.38 to 7.32 (p*K*_a1_ for RuL_3_), while further organization of the eight RuL_3_ motifs into a nanocage comprising six Pd^2+^/Pt^2+^ leads to a p*K*_a_ shift of more than five units down to p$K_{{\rm{a}}1{\rm{av}}}^{{\rm{Pd}}}$ = 4.59 (MOC-16) and 4.16 (MOC-39). This remarkable p*K*_a_ perturbation induced by highly positive charges [51] imparts prominent Brønsted acidity to nanocages that is comparable to acetic acid (p*K*_a _= 4.76), able to mediate an acid–base equilibrium via imidazole deprotonation. Since high charges are distributed on the cage wall, protons are prone to partial release into extrinsic media to balance electrostatic interactions. Our computational effort gains insight into the origin of this trend (Figs S10–S12) through exploration of the nanocage ionization activity in water via the calculation of the Gibbs free energy (Δ*G*_r_) of consecutive deprotonation processes. Dissociation of the first four protons from a nanocage is thermodynamically favorable (Δ*G*_1__–__4_ < 0), in agreement with the experimental observation that an aqueous solution of 1.0 mM MOC-16 is acidic (pH ≈2.5, approximate to the release of three protons per cage). Moreover, the prediction of the most preferential location of released protons indicates that proton transfer to H_2_O molecules outside the cage is more energetically favorable than proton transfer localized inside the cage-confined nanospace (Δ*G*_out_ = −42.52 kJ/mol versus Δ*G*_in_ = −29.17 kJ/mol), rationalizing the charge-induced deprotonation to generate solution nanocavities with heterogeneity of the intrinsic basicity and extrinsic acidity in a bulk solution. Both nanocages display outstanding robustness against acid (Fig. S13), retaining cage integrity even after acidifying extrinsic media to pH 1.5 for MOC-16 by adding 50 equiv. HOTf (CF_3_SO_3_H, equivalent to the amount per cage) or to pH 1.0 for MOC-39 by adding 200 equiv. HOTf. The Pt cage shows even better acid tolerance than the Pd cage, both exhibiting strong capacity to buffer the acid–base equilibrium via multiple imidazole-N/NH tautomerization.

**Figure 2.**
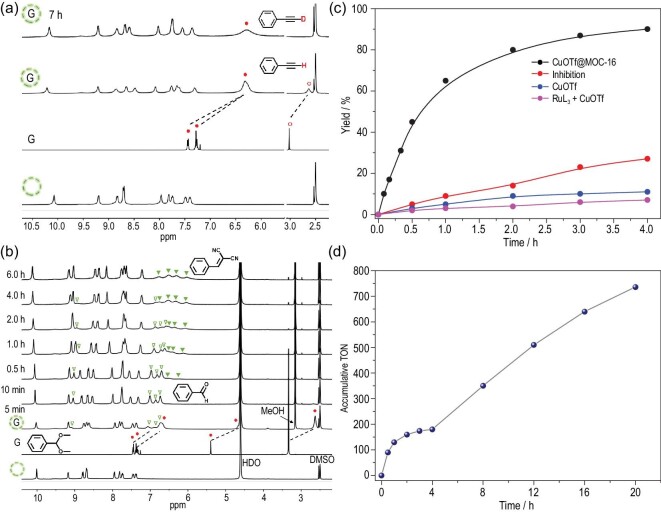
Multiple catalysis in the open-cage solution. (a) Deuteration of phenylacetylene (denoted by G) through encapsulation inside MOC-16 (represented by a dashed circle) in D_2_O–DMSO-*d*_6_ (v : v = 10 : 1, pD 2.5) media for 7 h at r.t. Red dots denote protons on phenylacetylene and red circles denote H–C_sp_. (b) ^1^H NMR *in situ* monitoring of acid/base-catalyzed one-pot cascade reaction of benzaldehyde dimethyl acetal (red dots) and malononitrile in the presence of MOC-16 (400 MHz, 300 K, D_2_O : DMSO-*d*_6_ = 10 : 1, v/v, pD 2.5). Hollow green triangles represent the hydrolysis product (aldehyde), and solid green triangles represent the final condensation product. (c) Catalytic kinetics of A^3^ coupling of 4-BrPhCHO, PhNH_2_ and phenylacetylene in the presence of 0.5 mol% CuOTf@MOC-16, showing the rate acceleration and competing guest inhibition by 1,1^′^-diacetyl ferrocene. (d) Accumulative TON of A^3^ coupling of 4-BrPhCHO, PhNH_2_ and phenylacetylene for five successive recycling reactions with the addition of aliquots of substrates (200 equiv.) at 6 h intervals after extraction of the product.

The formation of basic cage nanocavities in an acid solution with host–guest electrostatic interactions imposed by highly positive charges on a nanocage has been revealed by H/D-exchange experiments for direct C_sp_–H activation and deuteration under acidic conditions. Deuterated terminal alkynes are usually obtained via an acetylide utilizing strong bases (e.g. organolithium, Grignard reagent or Na metal) in aprotic solvents [52] and seldom in aqueous solutions, which rarely dissolve alkynes. Astonishingly, MOC-16/39 in aqueous media (pD ≈2.5) can easily trap alkynes to promote H abstraction and H/D exchange without the aid of any extraneous bases. The addition of phenylacetylene into MOC-16 solution results in an immiscible mixture, which quickly turns transparent, evidently due to the inclusion of phenylacetylene by MOC-16 via phase transfer. Compared to free phenylacetylene, the proton resonances of a trapped guest move upfield owing to the cage shielding effect, and that of H–C_sp_ gradually disappears after being kept at room temperature (r.t.) (Fig. 2a). GC–MS analysis reveals a deuterated product (*m*/*z*: 103, M^+^) with the efficient deuteration of phenylacetylene (98%). D_2_O serves as the deuterium source, and an inverse process is observed when treating the deuterated product in H_2_O–DMSO-*d*_6_ (Fig. S14). This observation suggests that a dynamic and reversible H/D-exchange process occurs inside the MOC-16 nanocavities even under acidic conditions, indicating that the weak acidity of phenylacetylene (p*K*_a_ = 23) is significantly enhanced by encapsulation owing to basic solution nanospaces and strong electrostatic interactions to stabilize the acetylide transition state. Similar deuteration of ethynylcyclohexane is observed with a slower deuteration rate for 40% H/D exchange at r.t. over 24 h (Fig. S15) due to the less effective host–guest compatibility of the nonaromatic cyclohexane ring. These results verify the formation of an acidic open-cage solution containing basic MOC-16 confined nanospaces with the supramolecular cage effect for the inclusion of insoluble alkynes via phase transfer and consequent C_sp_–H activation and deuteration.

**Figure 3.**
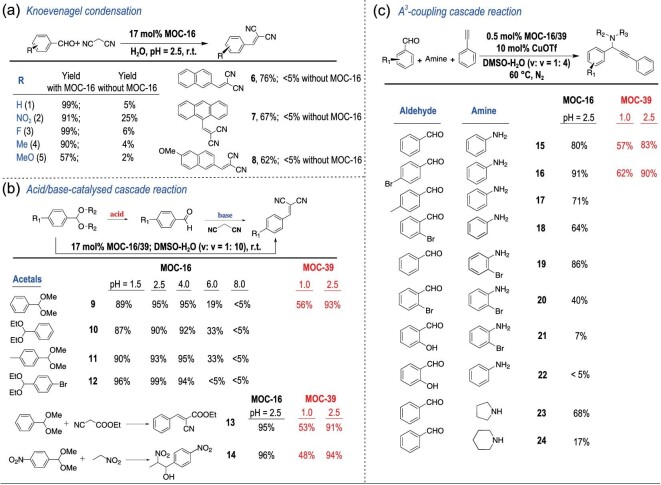
Substrate scope of cage-confined catalysis. The yields were determined by ^1^H NMR using mesitylene as the internal standard. Reaction conditions: (a) H_2_O (3.0 mL), 12 h. (b) DMSO (0.3 mL) + H_2_O (3.0 mL), 12 h. (c) DMSO (0.1 mL) + H_2_O (0.4 mL), 1 day.

The first cage-confined catalysis based on the open-cage solution of MOC-16 is tested for Knoevenagel condensation [47], which is known to start from deprotonation of the activated methylene for a nucleophilic addition to a carbonyl group, involving the formation of an anionic transition state usually catalyzed by a base. This condensation is promoted by MOC-16 even in acidic water environments for various aromatic aldehydes with malononitrile (**1–8**, Fig. 3a). Excellent yields are obtained for benzaldehyde and its derivatives, except for the methoxyl group (57%), and moderate yields are obtained for 2-naphthaldehydes (**6**, **8**) and anthracene-9-carbaldehyde (**7**). Under a similar acidic condition without MOC-16, little conversion can be observed, clearly demonstrating a supramolecular cage effect for C–H activation and oxyanion intermediate stabilization by the basic and positively charged cage-confined nanospaces in an acidic open-cage solution. The tests with low loading of 0.5 and 2 mol% MOC-16 for representative H/D exchange and Knoevenagel condensation under similar conditions indicate that the catalytic reactions are well processed with continuous increase of TONs along the reaction times, verifying the effectiveness of the cage-confined catalysis for these chemical transformations (Fig. S16).

To fully utilize the amphoteric nature of open-cage solutions containing MOC-16 nanocavities with intrinsic basicity and extrinsic acidity, we further test one-pot acid/base-catalyzed cascade reactions involving two steps: (i) the hydrolysis of acetal derivatives to aldehydes in an extrinsic acidic solution and (ii) the nucleophilic addition of the carbon anion of malononitrile to aldehydes to complete the Knoevenagel condensation inside the basic solution nanocavities (Fig. 3b). As seen from the reaction process monitored by *in situ*^1^H NMR (Fig. 2b), the hydrolysis of benzaldehyde dimethyl acetal finishes quickly (10 min), producing benzaldehyde to be encapsulated into MOC-16. The subsequent Knoevenagel condensation proceeds slowly, leading to targeted benzylidenemalononitrile (**9**) inside the cage in 6 h. Similar cascade reactions are smoothly performed for other acetal derivatives to afford products **10–12** (Figs S17–S19), evidently proving the dual functions of acidic open-cage solutions containing basic cage-confined nanospaces, namely, extrinsic Brønsted acidity in a bulk solution facilitating acid-catalyzed acetal hydrolysis, while intrinsic basicity in cage-confined nanospaces promotes Knoevenagel condensation. To explore the capacity of MOC-16 to generate robust solution nanospaces for acid–base amphoteric catalysis, we conduct these cascade reactions at different pH values (1.5–8.0) with or without MOC-16 (Table S10). All substrates afford excellent condensation yields in the presence of MOC-16 in a pH range of 1.5–4.0, suggesting a strong ability of nanocages to mediate acid–base equilibrium to maintain intrinsic basicity. In particular, the second-step Knoevenagel condensation of the cascade reaction is immune to extrinsic acid in the presence of MOC-16, even after strengthening the acidity to pH 1.5 by adding 50 equiv. HOTf. For comparison, when the pH value of the extrinsic solution is tuned by KOH to nearly neutral or basic conditions (pH = 6.0 and 8.0), the cascade reactions are greatly suppressed or shut off because of the inhibition of the first-step acetal hydrolysis. Moreover, the control experiments without MOC-16 produce few condensation products at pH 4.0, while under more basic or acidic conditions, the cascade reactions are completely suppressed at either the first or second step, respectively. This convenient one-pot acid/base catalytic protocol has also been extended to cascade reactions between benzaldehyde dimethyl acetal and ethyl 2-cyanoacetatein to form **13** and a hydrolysis–Henry cascade reaction to form **14**, both giving excellent yields. Note that two chiral carbon centers are generated in product **14**, which will present a pair of diastereomers. The ^1^H NMR spectrum of **14** reveals the formation of equal amounts of the diastereomers even when chiral Δ-MOC-16 is applied (Figs S20 and S21), indicating that no diastereoselectivity is induced by cage catalysis, which is in agreement with our previous observation that enantiopure Δ/Λ-MOC-16 is effective in resolving atropisomeric compounds but ineffective in resolving racemic molecules based on the chiral carbon centers [53]. The above results demonstrate the extraordinary merit of cage-confined catalysis on the basis of an open-cage solution, where cage-defined nanospaces create heterogeneity in solution to provide a versatile synthetic protocol for one-pot amphoteric and related cascade reactions.

Finally, one-pot three-component A^3^-coupling reactions [54] are tested to discover further advantages of cage-confined catalysis in open-cage solution. The commercial CuOTf⋅toluene complex is known to be moisture sensitive and suitable only for A^3^-coupling reactions in organic media, and alkyne addition to imines in water is known to be slow since alkynes are rarely water-soluble [55]. In contrast, MOC-16, with 24 imine-N sites around open portals, can bind with Cu^+^ ions to easily transfer and stabilize the water-insoluble and -sensitive CuOTf in an aqueous solution, thus switching the heterogeneous catalysis to a homogeneous behavior and concentrating multiple metal catalysts in an ordered fashion on the nanocage wall [38], which is beneficial for green purposes and catalytic efficiency. Moreover, the hydrophobicity and inclusion capacity of cage-confined nanospaces are able to expedite the phase transfer of organic substrates into aqueous nanocavities, while products are expelled from nanocages due to the lack of size fitting after chemical transformation. Therefore, the cage-confined catalysis of the A^3^ coupling in open-cage solution of MOC-16 features auto-recycling processes, and a very low loading of 0.5 mol% MOC-16 with a 20-fold concentration of CuOTf (10 mol%) in an aqueous media at pH 2.5 is effective enough for A^3^ coupling (Fig. 3c). The A^3^-coupling outcome (**15–24**) is found to be closely related to the molecular structure of both aldehydes and amines with varied substituent characteristics and positions, suggesting a subtle influence of the substrate structure on the cage-confined catalysis with delicate host–guest interactions (see details in the Supplementary Information). A series of control experiments and kinetic studies are carried out by using three benzaldehydes with different substituents, including recyclability and competitive-guest inhibition tests (Fig. 2c and d; Figs S22–S27 and Tables S11–S13). Clearly, in acidic aqueous media, A^3^-coupling one-pot reactions occurring in CuOTf@MOC-16 nanocavities display good effectiveness, comparable to the catalytic performance of homogeneous reactions in toluene with the aid of additional NEt_3_ while much better than that of heterogeneous reactions using CuOTf or CuOTf + RuL_3_ in water and homogeneous reactions using CuOTf in toluene under neutral conditions. The rate acceleration of cage-confined catalysis is estimated to be *k*_(CuOTf@MOC-16)_/*k*_(CuOTf)_ ≈ 55 for benzaldehyde, 42 for 4-bromobenzaldehyde and 71 for 4-methylbenzaldehyde, which are inhibitable by a competing 1,1^′^-diacetyl ferrocene guest. Notably, the recyclability tests verify the lasting catalytic efficiency, where successive reactions with five aliquots of substrates each in 200 equiv. Figs S22–S27 give accumulative TONs of 642 for benzaldehyde, 526 for 4-methylbenzaldehyde and 736 for 4-bromobenzaldehyde. These results evidently demonstrate the advantages of cage-confined catalysis in open-cage solutions for one-pot three-component reactions: (i) benefiting two transition states (metal acetylide and iminium ion) by the amphoteric nature and supramolecular cage effect for the final nucleophilic reaction and (ii) transferring water-insoluble metal catalysts and substrates/products in and out of an aqueous solution for catalytic efficiency and turnover.

The nanocage MOC-39 assembled from more inert Pt–N bonds shows superior acid resistance for catalytic reactions under even harsh conditions, which has been tested by further adjusting the extrinsic aqueous media to pH 1.0 by the addition of 200 equiv. HOTf (Fig. 3; Table S14). Excellent catalytic performance is observed at pH 2.5, while at pH 1.0, MOC-39 still promotes moderate conversion in all cases. This unique property to resist strong acidity is valuable for catalytic applications, considering that the cage integrity is well retained under harsh conditions to display a supramolecular cage effect for cage-confined catalysis, while the intrinsic basicity is still maintained by high-charge-mediated imidazole–proton equilibrium to manage the heterogeneity of open-cage solutions.

## CONCLUSION

In this work, we introduce supramolecular catalysis into open-cage solutions, demonstrating the generation of cage-defined nanospaces from dissolvable open cages in solution, where the cage-confined catalysis with multiple purposes and merits can be accomplished with the aid of the supramolecular cage effect. The highly positive charges on MOC-16/39 can not only induce a biofunctional p*K*_a_ shift and proton equilibrium via imidazole-N/NH protonation and deprotonation to mediate solution heterogeneity, i.e. Brønsted intrinsic basicity and extrinsic acidity, but also impose host–guest electrostatic interactions for substrate H-abstraction and anionic transition-state stabilization. Moreover, the hydrophobic effect endows the cage nanospaces with a strong guest inclusion capacity, while the abundant imidazole-N coordinative sites enable binding, concentrating and stabilizing metal catalysts on the cage wall, facilitating the phase transfer of water-insoluble substrates and catalysts into aqueous media for green and efficient catalysis under mild conditions. Many useful nucleophilic and acid/base-catalyzed reactions can be readily achieved in unconventional ways without adding extraneous bases while avoiding a water atmosphere. More interestingly, base catalysis can be achieved in harsh acidic conditions. This cage-confined catalytic protocol can be applied to either elementary reactions or two- and three-component amphoteric cascade one-pot reactions [56], featuring an enzymatic sense with prominent rate enhancement, catalytic turnover, good efficiency and recyclability for a wide scope of substrates.

## METHODS

All details on syntheses, single-crystal structure determination, p*K*_a_ determination, catalytic and kinetic studies and molecular simulations are provided in the Supplementary Data.

## Supplementary Material

nwab155_Supplemental_Files
