# Supplementary material for: Acidic open-cage solution containing basic cage-confined nanospaces for multipurpose catalysis
Source: Natl Sci Rev. 2021 Aug 20;9(5):nwab155. doi: 10.1093/nsr/nwab155 (PMC9155638; doi:10.1093/nsr/nwab155)
Supplement: nwab155_Supplemental_Files [file nwab155_supplemental_files.zip › Li_Kang_SI-R3.pdf]

Supplementary Information

Acid open-cage solution containing basic cage-confined nanospaces for multiple catalysis

Kang Li[1,2,4], Kai Wu[1,4], Yan-Zhong Fan[1,4], Jing Guo[1], Yu-Lin Lu[1], Yuan-Fan Wang[1], Guillaume Maurin[3] and Cheng-Yong Su[1,*]

[1] MOE Laboratory of Bioinorganic and Synthetic Chemistry, Lehn Institute of Functional Materials, School of Chemistry, Sun Yat-Sen University, Guangzhou 510275, China
[2] School of Chemistry, South China Normal University, Guangzhou 510006, China
[3] Institut Charles Gerhardt Montpellier, Centre National de la Recherche Scientifique, École Nationale Supérieure de Chimie de Montpellier, Université de Montpellier, Montpellier 34095, France
[4] These authors contributed equally
Email: cesscy@mail.sysu.edu.cn

Contents

1. Materials and methods………………………………………………………………… S2
2. Synthesis and characterization…………………………………………………….... S2
2.1 Synthesis of L, $RuL_3(BF_4)_2$, MOCs-16/39 and MOC-16@CuOTf......................………… S2
2.2 Single-crystal structure determination of MOC-39……………………………………….. S4
3 Typical procedures for catalysis…………………………………………………….. S6
3.1 H/D-exchange of alkynes…………………………………………………………….. S6
3.2 Knoevenagel-condensation…………………………………………………………… S7
3.3 Acid/base-catalyzed cascade reactions……………………………………………….. S8
3.4 A[3]-coupling tandem reactions……………………………………………………… S9
4. pK_a determination by potentiometric titration and theoretical calculations……………………. S11
4.1 The basics of this method…………………………………………………………….. S11
4.2 pK_a determination of L, $RuL_3(BF_4)_2$ and MOCs-16/39…………………………………… S13
4.3 Theoretical calculations……………………………………………………………….. S20
4.4 Acid stability of MOCs-16/39………………………………………………………… S22
5. Detailed study of H/D-exchange and Knoevenagel-condensation…………………………….. S23
6. Detailed study of acid/base cascade catalysis………………………………………….. S24
7. Detailed study of A[3]-coupling cascade catalysis……………………………………….. S27
8. References……………………………………………………………………………. S32

1. Materials and methods

Unless otherwise noted, all starting reagents and solvents were used as commercially purchased without further purification. Nuclear magnetic resonance (NMR) spectra were recorded on a Bruker AVANCE III 400 (400 MHz) spectrometer and ^{1}H chemical shifts were quoted in parts per million (ppm) relative to the signals corresponding to the residual non-deuterated solvents or 0.0 ppm for tetramethyl silane(TMS). Single crystal X-ray diffraction data were collected on an Agilent SuperNova X-ray diffractometer using micro-focus X-ray sources (Cu-K_α, λ = 1.54184 Å). HRESI-TOF mass spectra were measured on Bruker maXis 4G. The data analyses of ESI-TOF mass spectra were processed on Bruker Data Analysis software and the simulations were performed on Bruker IsotopePattern software. GC-MS analyses were carried out by an Agilent 5975C inert XL Triple Axis MSD with 7890 GC. The pH values were measured by a Mettler Toledo pH meter.

2. Synthesis and characterization

2.1 Synthesis of L, $RuL_3(BF_4)_2$, MOC-16/39 and MOC-16@CuOTf

The synthesis of **L**, **$RuL_3(BF_4)_2$** and **MOC-16** was according to our previously reported literature without further modification. (*1*)

Synthesis of MOC-39

$RuL_3(BF_4)_2$ (117 mg, 0.1 mmol) was dissolved in 4 mL DMSO with stirring, mixed with 3 eq of HBF_4 or HOTf, and then $[Pt(MeCN)_4](OTf)_2$ (99 mg, 0.15 mmol) in 1 mL of DMSO was added to above mixture. The solution was heated at 110 °C for 8 h. After cooling down the solution to r.t., 100 mL of ethyl acetate was added and the reddish orange precipitate was collected and centrifugated. After washing with ethyl acetate for three times, the powder was dried under vacuum to yield 155 mg cage solid, yield 95%. ^{1}H NMR (400 MHz, DMSO-*d₆* : D_2O = 1:5 v/v, 298 K): δ 10.02 (s, 24H), 9.13 (d, 24H), 8.78 (d, 24H), 8.69 (d, 48H), 7.94 (d, 24H), 7.81 (d, 24H), 7.75 (d, 24H), 7.46 (d, 24H), 7.40 (d, 24H). HRESI-MS: *m/z* calcd for $C_{432}H_{249}N_{123}O_9Pt_6Ru_8$ {[(MOC-39)-15H$^+$+3NO$_3^-$]$^{+10}$} 928.6304, found 928.6327; *m/z* calcd for $C_{432}H_{251}N_{125}O_{15}Pt_6Ru_8$ {[(MOC-39)-13H$^+$+5NO$_3^-$]$^{+10}$} 941.2296, found 941.2239; *m/z* calcd for $C_{432}H_{252}N_{126}O_{18}Pt_6Ru_8$ {[(MOC-39)-12H$^+$+6NO$_3^-$]$^{+10}$} 947.5291, found 947.5219; *m/z* calcd for $C_{432}H_{248}N_{123}O_9Pt_6Ru_8$ {[(MOC-39)-16H$^+$+3NO$_3^-$]$^{+9}$} 1031.6996, found 1031.6931; *m/z* calcd for $C_{432}H_{250}N_{125}O_{15}Pt_6Ru_8$ {[(MOC-39)-14H$^+$+5NO$_3^-$]$^{+9}$} 1045.6986, found 1045.6946.

Preparation of MOC-16@CuOTf

50.0 mg of MOC-16 (0.004 mmol) was dissolved in a mixture solution of DMSO and H_2O (0.3 mL/3.0 mL). Then, the water-insoluble Cu(I) triflate toluene complex (0.080 mmol) was introduced to the solution and kept for 1 h under stirring at room temperature. Afterward, a transparent solution was obtained as MOC-16@CuOTf. ^{1}H NMR

(400 MHz, DMSO-d_6 : D_2O = 1:10 v/v, 298 K): δ 10.16 (s, 24H), 9.29 (d, , J = 5.2 Hz, 24H), 8.92 (d, J = 7.6 Hz, 24H), 8.80 (d, J = 8.0 Hz, 48H), 8.06 (d, J = 4.8 Hz, 24H), 7.91 (d, J = 4.4 Hz, 24H), 7.83 (t, J = 6.4 Hz, 24H), 7.58 (t, J = 7.6 Hz, 24H), 7.49 (t, J = 6.8 Hz, 24H).

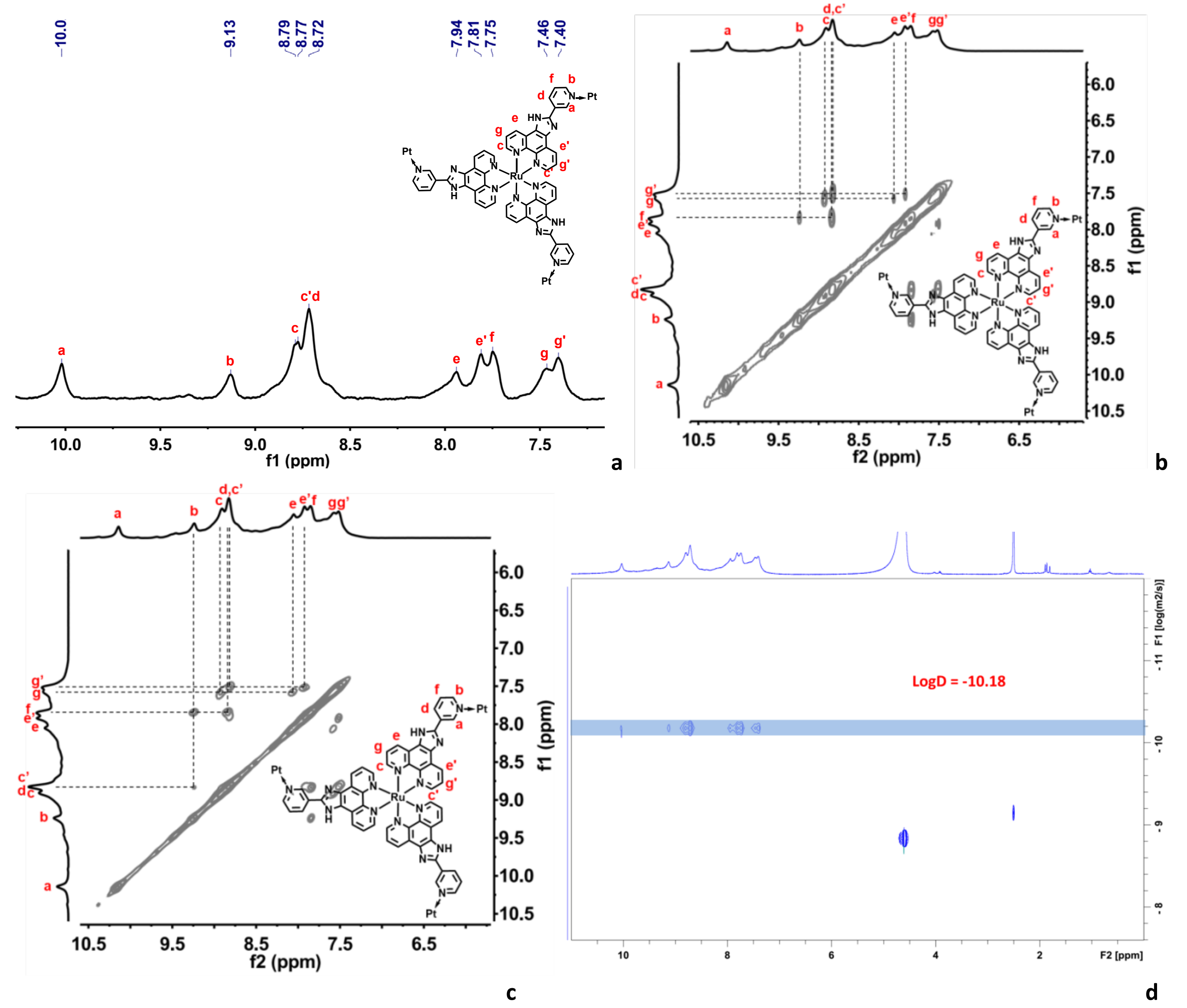

Supplementary Figure 1. (a) ^1H NMR spectrum, (**b**) ^1H-^1H COSY spectrum, (**c**) ^1H-^1H NOESY spectrum and (**d**) ^1H DOSY spectrum of MOC-39 in DMSO-d_6-D_2O (v: v = 1: 5).

2.2 Single-crystal structure determination of MOC-39

Single-crystals of MOC-39 suitable for single-crystal X-ray diffraction were obtained by diffusion of ethyl ether into a MeCN solution of MOC-39 (BF_4^- anions) and R-BINOL for 2 weeks. A red single-crystal was carefully picked, coated with paratone oil and attached to a glass fiber, which was inserted in a stainless steel stick. The crystal was quickly mounted to the Agilent Gemini S Ultra CCD Diffractometer with the Enhance X-ray Source of Cu radiation (λ = 1.54184 Å) using the ω-scan technique. The structure was solved by direct methods and refined by full-matrix least squares against F^2 using the SHELXL programs (*2*). Hydrogen atoms were placed in geometrically calculated positions and included in the refinement process using riding model with isotropic thermal parameters: Uiso(H) = 1.2 Ueq(-CH). All the electrons of disordered solvent molecules which cannot be determined are removed by SQUEEZE routine of PLATON program (*3*). Crystal and refinement parameters are listed in Table S1, and selected bond lengths (Å) and angles (°) are listed in Table S2.

Supplementary Table 1. Crystal data and structure refinement for MOC-39.

Identification code	**MOC-39**
Empirical Formula	$C_{512} H_{320} N_{120} O_8 Pt_6 Ru_8$
Formula weight	10259.96
T/K	150(2)
Crystal system	Tetragonal
Space group	*I*422
a/Å	32.4756(9)
b/Å	32.4756(9)
c/Å	38.0944(15)
α/°	90
β/°	90
γ/°	90
Volume/Å^3	40177(3)
Z	2
ρ_{calc}, g/cm^3	0.848
μ/mm^{-1}	3.414
Goodness-of-fit	1.011
R_1 [I≥2σ (I)]	0.0618
wR_2 (all data)	0.2145
CCDC No.	1990764
Data/restraints/parameters	16486 / 212 / 679
Flack value	0.088(18)

Supplementary Table 2. Selected bond lengths (Å) and angles (°) for MOC-39.

Pt(2)-N(15)	1.982(8)	Pt(1)-N(10)	1.919(19)
Pt(2)-N(15)[1]	1.982(8)	Ru(1)-N(1)	2.028(12)
Pt(2)-N(5)[2]	2.00(2)	Ru(1)-N(6)	2.030(18)
Pt(2)-N(5)[3]	2.00(2)	Ru(1)-N(11)	2.031(10)
Pt(1)-N(10)[3]	1.919(19)	Ru(1)-N(12)	2.041(19)
Pt(1)-N(10)[4]	1.919(19)	Ru(1)-N(2)	2.043(11)
Pt(1)-N(10)[5]	1.919(19)	Ru(1)-N(7)	2.05(2)
N(15)-Pt(2)-N(15)[1]	179.8(18)	N(6)-Ru(1)-N(11)	94.6(6)
N(15)-Pt(2)-N(5)[2]	91.0(6)	N(1)-Ru(1)-N(12)	90.0(7)
N(15)[1]-Pt(2)-N(5)[2]	89.0(13)	N(6)-Ru(1)-N(12)	171.5(8)
N(15)-Pt(2)-N(5)[3]	89.0(6)	N(11)-Ru(1)-N(12)	77.7(6)
N(15)[1]-Pt(2)-N(5)[3]	91.0(13)	N(1)-Ru(1)-N(2)	77.5(7)
N(5)[2]-Pt(2)-N(5)[3]	177.3(11)	N(6)-Ru(1)-N(2)	90.7(6)
N(10)[3]-Pt(1)-N(10)[4]	174.2(10)	N(11)-Ru(1)-N(2)	171.8(6)
N(10)[3]-Pt(1)-N(10)[5]	89.85(5)	N(12)-Ru(1)-N(2)	97.4(7)
N(10)[4]-Pt(1)-N(10)[5]	89.85(5)	N(1)-Ru(1)-N(7)	173.1(6)
N(10)[3]-Pt(1)-N(10)	89.86(5)	N(6)-Ru(1)-N(7)	80.3(7)
N(10)[4]-Pt(1)-N(10)	89.85(5)	N(11)-Ru(1)-N(7)	89.2(5)
N(10)[5]-Pt(1)-N(10)	174.2(10)	N(12)-Ru(1)-N(7)	95.7(8)
N(1)-Ru(1)-N(6)	94.5(7)	N(2)-Ru(1)-N(7)	97.9(7)
N(1)-Ru(1)-N(11)	95.8(5)		

[1]y,x,-z; [2]x,-y-1,-z; [3]-y-1,x,z; [4]y,-x-1,z; [5]-x-1,-y-1,z; [6]-y-3/2,-x-3/2,-z-1/2

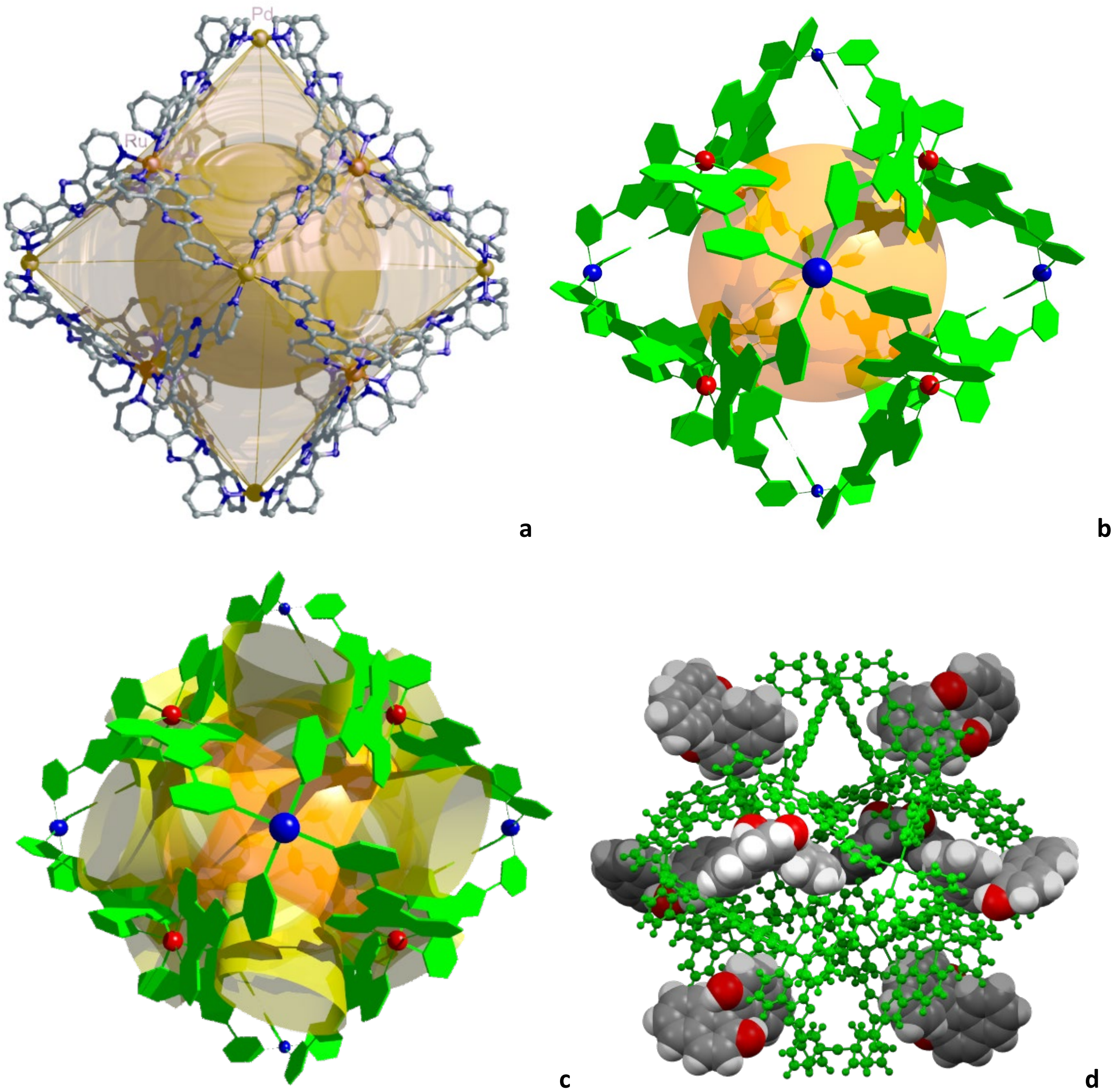

Supplementary Figure 2. Single-crystal structures of MOC-16/39. (**a**) Stick-and-ball cage structure showing octahedral cavity. (**b**) Cage with aromatic planar ligands and interior. (**e**) Cage with 12 open rhombic window portals. (**d**) Capture of eight 4,4-Binol guests inside the portals.

3. Typical procedures for catalysis

3.1 H/D exchange of alkynes

Typically, to a mixed solution of MOC-16 (50.0 mg, 0.004 mmol) in DMSO and D_2O (0.3 mL/ 3.0 mL) was added phenylacetylene (2.7 µL, 0.024 mmol) in one portion. The solution was kept for stirring at room temperature for 7 h and extracted by $CDCl_3$ (800 µL) for NMR analysis. ^1H NMR (400 MHz, $CDCl_3$, 298 K): δ 7.53 (d, J = 8.0 Hz, 2H),

7.35 (m, 3H); EI-MS *m/z* 103 [M^+]. The low catalyst-loading catalysis was carried out under similar conditions in the presence of 2 and 0.5 mol% MOC-16.

3.2 Knoevenagel-condensation catalysis

To the mixed solution of MOC-16 (0.004 mmol) in DMSO (0.3 mL) and H_2O (3.0 mL) was added substrates of aldehyde (0.024 mmol) and malononitrile (1.6 mg, 0.024 mmol) in one portion. The solution was stirred at room temperature for 12 h, then extracted with diethyl ether (3.0 mL× 3). The combined organic phase was evaporated under vacuum to obtain the desired product. The low catalyst-loading catalysis was carried out under similar conditions in the presence of 2 and 0.5 mol% MOC-16.

Physical data of condensation products:

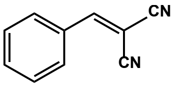

(R1)

^{1}H NMR (400 MHz, $CDCl_3$, 298 K): δ 7.91 (d, *J* = 8.4 Hz, 2H), 7.79 (s, 1H), 7.64 (t, *J* = 7.6 Hz, 1H), 7.55 (t, *J* = 7.6 Hz, 2H). EI-MS *m/z* 154 [M^+].

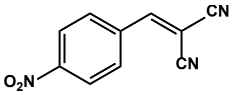

(R2)

^{1}H NMR (400 MHz, $CDCl_3$, 298 K): δ 8.39 (d, *J* = 8.8 Hz, 2H), 8.08 (d, *J* = 8.8 Hz, 2H), 7.91 (s, 1H). EI-MS *m/z* 199 [M^+].

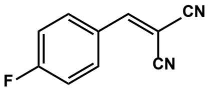

(R3)

^{1}H NMR (400 MHz, $CDCl_3$, 298 K): δ 7.96 (d, *J* = 8.8 Hz, 2H), 7.75 (s, 1H), 7.24 (d, *J* = 8.8 Hz, 2H). EI-MS *m/z* 172 [M^+].

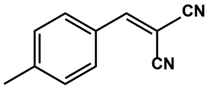

(R4)

^{1}H NMR (400 MHz, $CDCl_3$, 298 K): δ 7.81 (d, *J* = 7.6 Hz, 2H), 7.73 (s, 1H), 7.34 (d, *J* = 8.0 Hz, 2H), 2.28 (s, 3H). EI-MS *m/z* 168 [M^+].

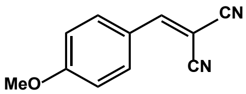

(R5)

^{1}H NMR (400 MHz, $CDCl_3$, 298 K): δ 7.91 (d, *J* = 8.8 Hz, 2H), 7.66 (s, 1H), 7.01 (d, *J* = 8.8 Hz, 2H), 3.92 (s, 3H). EI-MS *m/z* 184 [M^+].

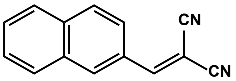

(R6)

^{1}H NMR (400 MHz, $CDCl_3$, 298 K): δ 8.29 (s, 1H), 8.07 (d, J = 8.8 Hz, 1H), 7.98-7.93 (m, 2H), 7.92-7.87 (m, 2H), 7.68 (t, J = 6.8 Hz, 1H), 7.61 (t, J = 7.0 Hz, 1H). EI-MS m/z 204 [M^+].

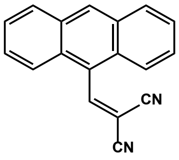

(R7)

^{1}H NMR (400 MHz, $CDCl_3$, 298 K): δ 8.95 (s, 1H), 8.64 (s, 1H), 8.07 (d, J = 6.8 Hz, 2H), 7.92 (d, J = 8.4 Hz, 2H), 7.68 (t, J = 6.8 Hz, 2H), 7.57 (t, J = 8.0 Hz, 2H). HRESI-MS: Calcd. 255.0922 [$(M+H)^+$]. Found: 255.0939.

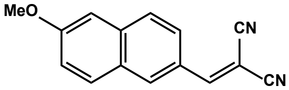

(R8)

^{1}H NMR (400 MHz, $CDCl_3$, 298 K): δ 8.20 (s, 1H), 8.05 (d, J = 9.2 Hz, 1H), 7.84 (d, J = 6.4 Hz, 2H), 7.83 (s, 1H), 7.28-7.21 (m, 1H), 7.20-7.15 (m, 1H), 3.97 (s, 3H). HRESI-MS: Calcd. 257.0691 [$(M+Na)^+$]. Found: 257.0707.

3.3 Acid/base-catalyzed cascade reactions

To the mixed solution of MOC-16 (0.004 mmol) in DMSO (0.3 mL) and H_2O (3.0 mL) was added substrates of aryl acetal (0.024 mmol) and malononitrile (1.6 mg, 0.024 mmol) / ethyl cyanoacetate (2.6 μl, 0.024 mmol) / nitroethane (10 μl, 0.120 mmol) in one portion. The solution was stirred at room temperature for 12 h, then extracted with diethyl ether (3.0 mL× 3). The combined organic phase was evaporated under vacuum to obtain the desired product.

Physical data of condensation products:

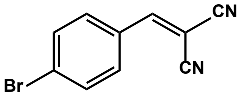

(R12)

^{1}H NMR (400 MHz, $CDCl_3$, 298 K): δ 7.77 (d, J = 8.4 Hz, 2H), 7.73 (s, 1H), 7.69 (d, J = 8.4 Hz, 2H). HRESI-MS: Calcd. 254.9534 [$(M+Na)^+$]. Found: 254.9553.

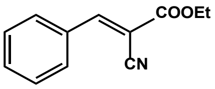

(R13)

^{1}H NMR (400 MHz, $CDCl_3$, 298 K): δ 8.26 (s, 1H), 7.99 (d, J = 7.6 Hz, 2H), 7.60-7.45 (m, 3H), 4.39 (q, J = 7.2 Hz, 2H), 1.40 (t, J = 7.2 Hz, 3H). HRESI-MS: Calcd. 224.0687 [$(M+Na)^+$]. Found: 224.0702.

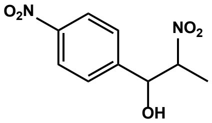

(R14)

^{1}H NMR (400 MHz, $CDCl_3$, 298 K): δ 8.25 (dd, J_1 = 8.4 Hz, J_2 = 3.6 Hz, 4H), 7.59 (t, J = 7.6 Hz, 4H), 5.52 (d, J = 3.6 Hz, 1H), 5.15 (d, J = 8.8 Hz, 1H), 4.84-4.64 (m, 2H), 1.47 (d, J = 7.2 Hz, 3H), 1.36 (d, J = 6.8 Hz, 3H). HRESI-MS: Calcd. 249.0487 [$(M+Na)^+$]. Found: 249.0503.

3.4 A^3-coupling tandem reactions

The Cu(I) triflate toluene complex (10 mg ,0.02 mmol) was added to the DMSO/H_2O solution (0.1 mL + 0.4 mL) of MOC-16 (12 mg, 0.001 mmol) and stirred for 1 h at r.t. to form an homogeneous solution. Then, the substrates of aldehyde (0.2 mmol), aniline (0.24 mmol), and phenylacetylene (0.3 mmol) were stepwise introduced to the above solution. The reaction mixture was stirred at 60 ℃ for 6 h under nitrogen atmosphere. After completion of the reaction, the mixture was extracted with diethyl ether (1.0 mL × 3). The combined organic phase was evaporated under vaccum to obtain the product.

Compared with a benchmark A^3-coupling of benzaldehydes, aniline and PA with good yield (**15**, 80%), electron-donating methyl substitution on *para*-position of benzaldehyde causes a lowered yield of 71% (**17**), but electron-withdrawing Br-substitution leads to improved conversion to 91% (**16**). By contrast, Br-substitution on *ortho*-position of either aniline or benzaldehyde only gives varied yields 40-86% (**18-20**), while the hydroxyl substituent results in ineffective A^3-coupling (**21**, **22**). Moreover, the nonaromatic amine of pyrrolidine provides a moderate conversion of 68% (**23**), while piperidine offers very low yield of 17% (**24**).

Physical data of A^3-coupling products:

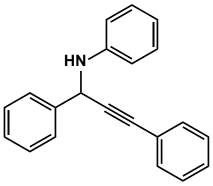

(R15)

^{1}H NMR (400 MHz, $CDCl_3$, 298 K): δ 7.69 (d, J = 8.0 Hz, 2H), 7.46-7.20 (m, 10H), 6.85-6.78 (m, 3H), 5.53 (s, 1H), 4.19 (s, 1H); HRESI-MS: Calcd. 284.1439 [$(M+H)^+$]. Found: 284.1459.

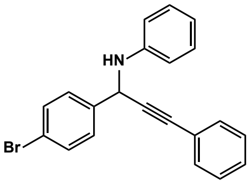

(R16)

^{1}H NMR (400 MHz, $CDCl_3$, 298 K): δ 7.54-7.48 (m, 4H), 7.42-7.37 (m, 2H), 7.29-7.25 (m, 3H), 7.22-7.17 (m, 2H), 6.78 (t, *J* = 8.0 Hz, 1H), 6.72 (d, *J* = 8.0 Hz, 2H), 5.44 (s, 1H), 4.12 (br, 1H); HRESI-MS: Calcd. 362.0544 [$(M+H)^+$].; found, 362.0559.

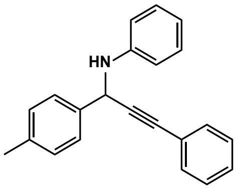

(R17)

^{1}H NMR (400 MHz, $CDCl_3$, 298 K): δ 7.52 (d, *J* = 8.0 Hz, 2H), 7.43-7.36 (m, 2H), 7.28-7.23 (m, 3H), 7.22-7.16 (m, 4H), 6.80-6.73 (m, 3H), 5.44 (s, 1H), 3.80 (br, 1H), 2.35 (s, 3H); HRESI-MS: Calcd. 298.1596 [$(M+H)^+$]. Found: 298.1611.

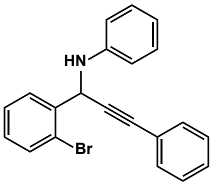

(R18)

^{1}H NMR (400 MHz, $CDCl_3$, 298 K): δ 7.80 (d, *J* = 7.8 Hz, 1H), 7.61 (d, *J* = 7.9 Hz, 1H), 7.36-7.32 (m, 2H), 7.30–7.22 (m, 1H), 7.22-7.10 (m, 6H), 6.77 (t, *J* = 7.4 Hz, 1H), 6.72 (d, *J* = 7.8 Hz, 2H), 5.78 (s, 1H). HRESI-MS: Calcd. 362.0544 [$(M+H)^+$]. Found: 362.0559.

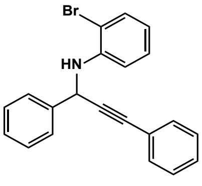

(R19)

^{1}H NMR (400 MHz, $CDCl_3$, 298 K): δ 7.66 (d, *J* = 7.6 Hz, 2H), 7.45 (d, *J* = 8.0 Hz, 1H), 7.43-7.38 (m, 4H), 7.35 (d, , *J* = 7.6 Hz, 1H), 7.30-7.25 (m, 3H), 7.18 (t, *J* = 8.0 Hz, 1H), 6.89 (d, *J* = 8.0 Hz, 1H), 6.63 (t, *J* = 7.6 Hz, 1H), 5.53 (s, 1H). HRESI-MS: Calcd. 362.0544 [$(M+H)^+$]. Found: 362.0561.

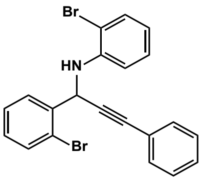

(R20)

^{1}H NMR (400 MHz, $CDCl_3$, 298 K): δ 7.92-7.87 (m, 1H), 7.78 (d, *J* = 6.4 Hz, 1H), 7.48 (d, *J* = 6.8 Hz, 2H), 7.41-7.37 (m, 2H), 7.22-7.11 (m, 3H), 6.74 (d, *J* = 8.4 Hz, 2H), 6.66-6.56 (m, 2H), 5.81 (s, 1H). HRESI-MS: Calcd. 441.9629 [$(M+H)^+$]. Found: 441.9643.

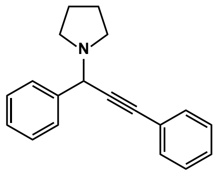

(R23)

^{1}H NMR (400 MHz, $CDCl_3$, 298 K): δ 7.68-7.61(m, 2H), 7.59-7.53(m, 2H), 7.42-7.36(m, 2H), 7.35-7.29(m, 4H), 4.91(s, 1H), 2.75-2.67(m, 4H), 1.85-1.80(m, 4H); HRESI-MS: Calcd. 262.1596 [$(M+H)^+$]. Found: 262.1613.

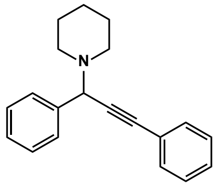

(R24)

^{1}H NMR (400 MHz, $CDCl_3$, 298 K): δ 7.69-7.62(m, 2H), 7.55-7.49(m, 2H), 7.40-7.26(m, 6H), 4.81(s, 1H), 2.62-2.52(m, 4H), 1.72-1.51(m, 4H), 1.51-1.40(m, 2H); HRESI-MS: Calcd. 276.1752 [$(M+H)^+$]. Found: 276.1766.

4. pK_a determination by potentiometric titration and theoretical calculations

4.1 The basics of this method

In a typical procedure of potentiometric titration, a certain volume of reagent (base solution) is added stepwise to a solution of analyte. The change in potential upon titration is recorded by pH meter. The Bjerrum function is used to describe the relationship between the volume and potential (*4*):

$$\overline{n}_{\mathrm{H}} = f(V, [\mathrm{H}])$$

where $\overline{n}_{\mathrm{H}}$ is defined as the average number of associated protons per acid group.

For the monoacid (HA):

$$\overline{n}_{\mathrm{H}} = \frac{[\mathrm{HA}]}{c_{\mathrm{HA}}} = \frac{[\mathrm{HA}]}{[\mathrm{A}]+[\mathrm{HA}]} = \frac{[\mathrm{HA}]}{\frac{[\mathrm{HA}]K_{\mathrm{a}}}{[\mathrm{H}]}+[\mathrm{HA}]} = \frac{[\mathrm{H}]}{[\mathrm{H}]+K_{\mathrm{a}}} \qquad (1)$$

in which, [HA] and c_{HA} represent the real-time and starting concentration of monoacid, respectively. K_a is the dissociation constant.

The equation (1) is transformed to a logarithm form:

$$\mathrm{p}K_a = -\lg[\mathrm{H}] + lg\frac{\overline{n}_{\mathrm{H}}}{1-\overline{n}_{\mathrm{H}}} = \mathrm{pH} + lg\frac{\overline{n}_{\mathrm{H}}}{1-\overline{n}_{\mathrm{H}}} \qquad (2)$$

For the binary acid (H_2A):

$$\bar{n}_H = \frac{[HA]+2[H_2A]}{c_{H_2A}} = \frac{[HA]+2[H_2A]}{[A]+[HA]+[H_2A]} = \frac{\beta_1^H[H]+2\beta_2^H[H]^2}{1+\beta_1^H[H]+\beta_2^H[H]^2} \quad (3)$$

$$\frac{\bar{n}_H}{(1-\bar{n}_H)[H]} = \beta_1^H + \frac{(2-\bar{n}_H)[H]}{1-\bar{n}_H}\beta_2^H \quad (4)$$

where

$$\beta_1^H = \frac{[HA]}{[H][A]} = \frac{1}{K_{a2}}, \quad \beta_2^H = \frac{[HA][H_2A]}{[H]^2[HA][A]} = \frac{1}{K_{a1}K_{a2}} \quad (5)$$

From equation (5), the two pK_a *values* are deduced as follows:

$$pK_{a1} = -lg\frac{\beta_1^H}{\beta_2^H}, \quad pK_{a2} = -lg\frac{1}{\beta_1^H} \quad (6)$$

For the ternary acid (H_3A):

$$\bar{n}_H = \frac{[HA]+2[H_2A]+3[H_3A]}{c_{H_3A}} = \frac{[HA]+2[H_2A]+3[H_3A]}{[A]+[HA]+[H_2A]+[H_3A]} = \frac{\beta_1^H[H]+2\beta_2^H[H]^2+3\beta_3^H[H]^3}{1+\beta_1^H[H]+\beta_2^H[H]^2+\beta_3^H[H]^3} \quad (7)$$

after rearrangement,

$$\frac{\bar{n}_H}{(1-\bar{n}_H)[H]} = \beta_1^H + \frac{(2-\bar{n}_H)[H]}{1-\bar{n}_H}\beta_2^H + \frac{(3-\bar{n}_H)[H]^2}{1-\bar{n}_H}\beta_3^H \quad (8)$$

where

$$\beta_1^H = \frac{[HA]}{[H][A]} = \frac{1}{K_{a3}}, \beta_2^H = \frac{[HA][H_2A]}{[H]^2[HA][A]} = \frac{1}{K_{a2}K_{a3}}, \ \beta_3^H = \frac{[HA][H_2A][H_3A]}{[H]^3[H_2A][HA][A]} = \frac{1}{K_{a1}K_{a2}K_{a3}} \quad (9)$$

In practical data processing, through selection of suitable titration area, the equation (8) can be simplified as follows,

$$\frac{\bar{n}_H}{(1-\bar{n}_H)[H]} \approx \beta_1^H + \frac{(2-\bar{n}_H)[H]}{1-\bar{n}_H}\beta_2^H \quad (10)$$

Once the β_1^H and β_2^H are obtained through linear fitting according to equation (10), the equation (8) can be transformed to another linear function to fit the β_3^H,

$$\frac{\bar{n}_H-\beta_1^H(1-\bar{n}_H)[H]}{(2-\bar{n}_H)[H]^2} = \beta_2^H + \frac{(3-\bar{n}_H)[H]}{2-\bar{n}_H}\beta_3^H \quad (11)$$

Finally, from equation (9), the three pK_a *values* are deduced as follows:

$$pK_{a1} = -lg\frac{\beta_2^H}{\beta_3^H}, \ pK_{a2} = -lg\frac{\beta_1^H}{\beta_2^H}, \ pK_{a3} = -lg\frac{1}{\beta_1^H} \quad (12)$$

4.2 pK_a determination of L, $RuL_3(BF_4)_2$ and MOC-16/39

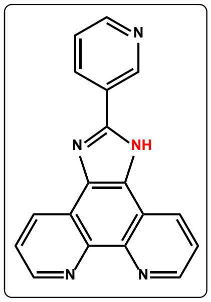

L

The monoacid (HA) model can be applied to the organic ligand (**L**) for pK_a determination because of only one NH motif containing in this structure.

Experimental procedure for L:

To a solution of **L** (4.90 mM, 25.00 mL H_2O + 25.00 mL DMSO, I = 50.00 mM) was added 0.10 mL of aqueous KOH (98.90 mM) under stirring for 5 min at each time. Then the pH value was recorded by pH meter (calibrated by standard buffer solution of 4.01 and 6.86 before used). The titration process was continued until the jump of the pH value of the solution, which means reaching the end point of the titration. The whole process was conducted at 25 ^oC under nitrogen atmosphere.

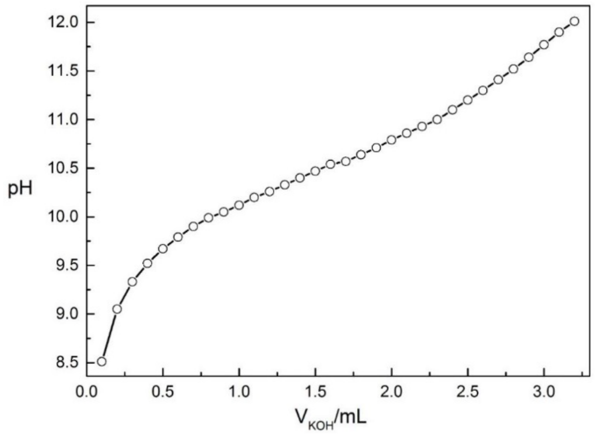

Supplementary Figure 3. The potentiometric titration curve of **L.**

Supplementary Table 3. Date processing using the equation (**1**) and (**2**)

V_{KOH}/mL	pH	$\bar{n}_H$	$\lg\frac{\bar{n}_H}{1-\bar{n}_H}$	pK_a	V_{KOH}/mL	pH	$\bar{n}_H$	$\lg\frac{\bar{n}_H}{1-\bar{n}_H}$	pK_a
0.00	6.14	1.00			1.20	10.26	0.55	0.09	10.35
0.10	8.51	0.96	1.38		1.30	10.33	0.52	0.03	10.36
0.20	9.05	0.92	1.06		1.40	10.40	0.49	-0.02	10.38
0.30	9.33	0.88	0.86		1.50	10.47	0.46	-0.07	10.40
0.40	9.52	0.84	0.72		1.60	10.54	0.43	-0.12	10.42
0.50	9.67	0.81	0.63	10.30	1.70	10.57	0.39	-0.19	10.38
0.60	9.79	0.77	0.52	10.31	1.80	10.64	0.36	-0.25	10.39
0.70	9.90	0.73	0.43	10.33	1.90	10.71	0.34	-0.29	10.42
0.80	9.99	0.70	0.37	10.36	2.00	10.79	0.32	-0.33	10.46
0.90	10.05	0.66	0.29	10.34	2.10	10.86	0.30	-0.37	10.49
1.00	10.12	0.62	0.21	10.33	2.20	10.93	0.29	-0.39	
1.10	10.20	0.59	0.16	10.36	2.30	11.00	0.28	-0.41	

After selecting the suitable area of $\bar{n}_H$ (0.30~0.63) to calculate the pK_a, we can finally obtain an average value of **10.38 (0.04)** for **L** in H_2O-DMSO (v: v = 1: 1) solution.

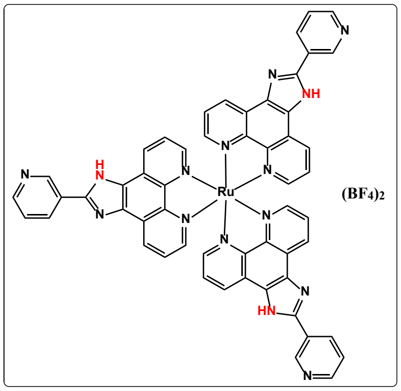

Metalloligand (RuL_3)

Experimental procedure for $RuL_3(BF_4)_2$:

To a solution of $RuL_3(BF_4)_2$ (1.70 mM, 25.00 mL H_2O + 25.00 mL DMSO, I = 50.00 mM) was added 0.05 mL of aqueous KOH (102.50 mM) under stirring for 5 min at each time. Then the pH value was recorded by pH meter (calibrated by standard buffer solution of 4.01 and 6.86 before used). The titration process was continued until the jump of the pH value of the solution, which means reaching the end point of the titration. The whole process was conducted at 25 °C under nitrogen atmosphere.

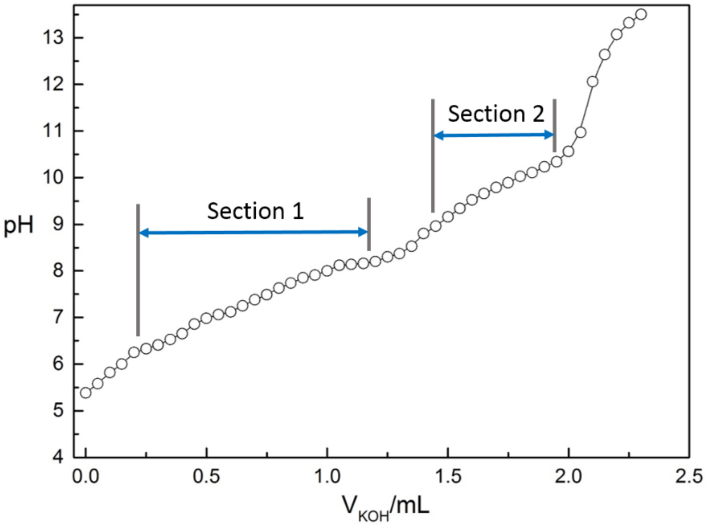

Supplementary Figure 4. The potentiometric titration curve of **RuL_3**

In principle, the ternary acid model (**H_3A**) can be applied to the $RuL_3(BF_4)_2$ for pK_a determination. Nevertheless, the titration curve of $RuL_3(BF_4)_2$ shows two distinct buffer area (**section 1** and **2**). According to the consumption of KOH quantity for neutralization, the **section 1** can be assigned to the overlap of the first and second proton dissociation processes of $RuL_3(BF_4)_2$ and **section 2** is assigned to the third proton dissociation process. Benefiting from this phenomenon, the binary acid model and monoacid model can be applied for the three pK_a values determination of $RuL_3(BF_4)_2$, respectively.

Supplementary Table 4. Data processing using the binary acid model for section 1.

V_{KOH}/mL	pH	$\bar{n}_H$	$\frac{(2-\bar{n}_H)[H]}{1-\bar{n}_H}$	$\frac{\bar{n}_H}{(1-\bar{n}_H)[H]}$
0.75	7.49	1.12		
0.80	7.63	1.06		
0.85	7.74	0.99		
0.90	7.85	0.93	2.16×10^{-7}	9.42×10^{8}
0.95	7.91	0.88	1.15×10^{-7}	5.96×10^{8}
1.00	8.00	0.82	9.33×10^{-8}	4.56×10^{8}
1.05	8.12	0.75	3.79×10^{-8}	3.96×10^{8}
1.10	8.14	0.69	3.52×10^{-8}	2.68×10^{8}
1.15	8.16	0.63	2.56×10^{-8}	2.46×10^{8}
1.20	8.20	0.57	2.10×10^{-8}	2.10×10^{8}

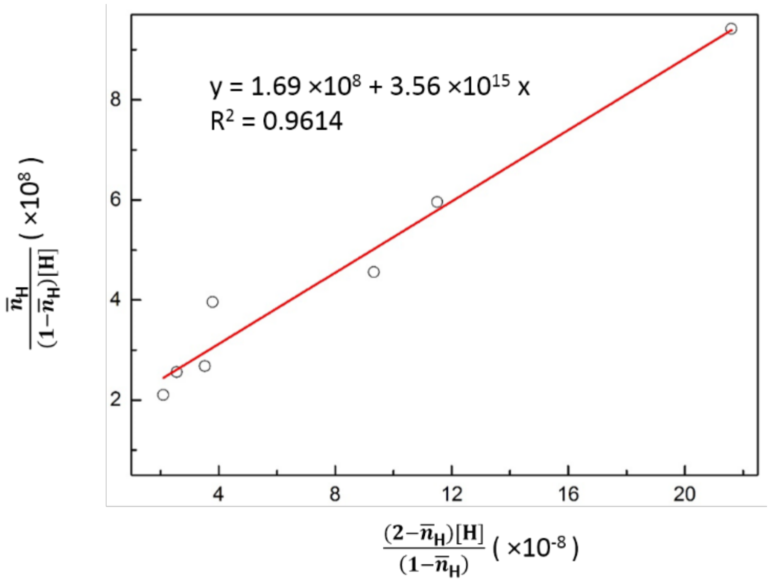

Supplementary Figure 5. Linear fitting of the data using equation (**4**)

The fitted linear function gives the values of β_1^H and β_2^H to be 1.69×10^8 and 3.56×10^{15}, respectively. According to equation (**6**), the first two pK_a values are determined to be:

pK_{a1} = 7.32 (0.04) , pK_{a2} = 8.23 (0.05)

Supplementary Table 5. Data processing using the monoacid model for section 2.

V_{KOH}/mL	pH	$\overline{n}_H$	$\lg\frac{\overline{n}_H}{1-\overline{n}_H}$	pK_a
1.45	8.96	0.94	1.19	10.15
1.50	9.16	0.89	0.91	10.07
1.55	9.34	0.83	0.69	10.03
1.60	9.52	0.78	0.55	10.07
1.65	9.66	0.73	0.43	10.09
1.70	9.79	0.68	0.33	10.12
1.75	9.89	0.63	0.23	10.12
1.80	10.03	0.59	0.16	10.19
1.85	10.11	0.54	0.07	10.18
1.90	10.23	0.51	0.03	10.25
1.95	10.34	0.48	-0.03	10.31

After selecting the suitable area of $\overline{n}_H$ (0.48~0.94) to calculate the pK_a, third value of **RuL_3** is determined to be **pK_{a3} = 10.14 (0.07)**.

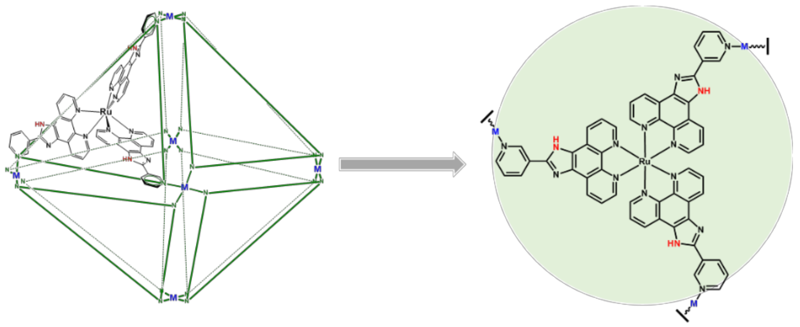

Supplementary Figure 6. Simplified model for pK_a measurement of MOC-16/39.

One MOC-16 or MOC-39 contains 24 subcomponents of imidazole group, which corresponding to a sum of 24 pK_a values (pK_{a1} to pK_{a24}). It is a tremendous difficulty for either experimental measurements or theoretical simulations concerning *ca.*1000 atoms on the MOC-16/39 framework. Alternatively, a simplified pK_a model may be used to handle this problem. Due to the high O_h symmetry of MOC-16/39 in solution, the 8 RuL_3 located on each octahedron facets are stereoscopically and chemically equivalent. So the pK_a model can be simplified as one subcomponent of RuL_3 that influenced by coordination of three Pd^{2+} to terminal pyridine at each corner (**Fig. S6**). After such simplification, we only need to determine three pK_a values that derived from the imidazole motif of RuL_3 (pK_{a1av}, p$K_{a2av,}$ pK_{a3av}). In this case, pK_{a1av} represents an average of pK_a values from pK_{a1} to pK_{a8}, pK_{a2av} refers to an average from pK_{a9} to pK_{a16}, and pK_{a3av} refers to an average from pK_{a17} to p$K_{a24.}$ Therefore, the ternary acid model can be applied for pK_a determination.

Experimental procedure for MOC-16/39:

To a solution of MOC-16/39 (0.17 mM, 25.00 mL H_2O + 25.00 mL DMSO, I = 50.00 mM) was added 0.05 mL of aqueous KOH (86.8 mM for MOC-16 or 96.2 mM for MOC-39) under stirring for 5 min at each time. Then the pH value was recorded by pH meter (calibrated by standard buffer solution of 4.01 and 6.86 before used). The titration process was continued until the jump of the pH value of the solution, which means reaching the end point of the titration. The whole process was conducted at 25 °C under nitrogen atmosphere. ***Note:*** In order to prevent the loss of protons during the preparation of MOC-16/39, the sample was synthesized in DMSO and prepared directly to the testing solution without isolation of product. Considering the extra acid may be introduced by $Pd(BF_4)_2$, the background influence was estimated and deducted at the beginning of the titration.

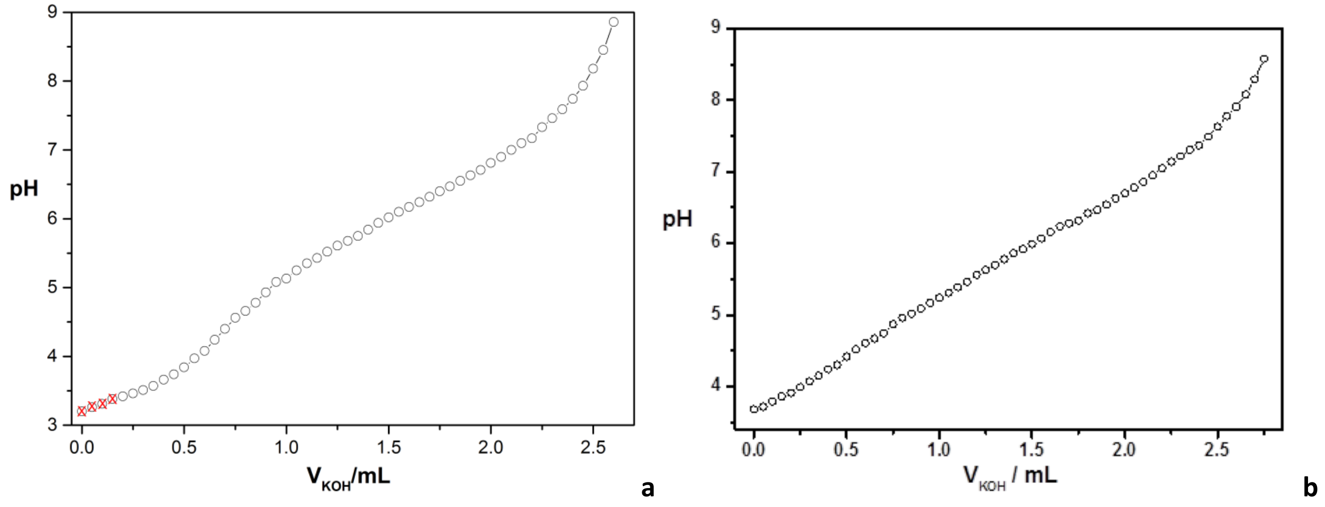

Supplementary Figure 7. The potentiometric titration curves of (**a**) MOC-16 and (**b**) MOC-39.

Supplementary Table 6. Data processing using the equation (**7**) with $\bar{n}_H$ ranging from 0.64 to 0.93 for MOC-16

V_{KOH} /mL	pH	$\bar{n}_H$	$\frac{(2-\bar{n}_H)[H]}{1-\bar{n}_H}$	$\frac{\bar{n}_H}{(1-\bar{n}_H)[H]}$
1.70	6.71	0.93	2.98 ×10^{-6}	6.81×10^{7}
1.75	6.81	0.87	1.35×10^{-6}	4.32×10^{7}
1.80	6.90	0.81	7.89×10^{-7}	3.38×10^{7}
1.85	7.00	0.76	5.17×10^{-7}	3.17×10^{7}
1.90	7.10	0.70	3.44×10^{-7}	2.94×10^{7}
1.95	7.17	0.64	2.55×10^{-7}	2.63×10^{7}

Supplementary Table 7. Data processing using the equation (**7**) with $\overline{n}_H$ ranging from 0.45 to 0.81 for MOC-39

V_{KOH} /mL	pH	$\overline{n}_H$	$\frac{(2-\overline{n}_H)[H]}{1-\overline{n}_H}$	$\frac{\overline{n}_H}{(1-\overline{n}_H)[H]}$
1.75	6.32	0.81	3.00×10^{-6}	8.90×10^{6}
1.80	6.42	0.75	1.90×10^{-6}	7.89×10^{6}
1.85	6.47	0.69	1.43×10^{-6}	6.56×10^{6}
1.90	6.54	0.63	1.07×10^{-6}	5.91×10^{6}
1.95	6.63	0.57	7.78×10^{-7}	5.66×10^{6}
2.00	6.70	0.51	6.08×10^{-7}	5.20×10^{6}
2.05	6.78	0.45	4.68×10^{-7}	4.93×10^{6}

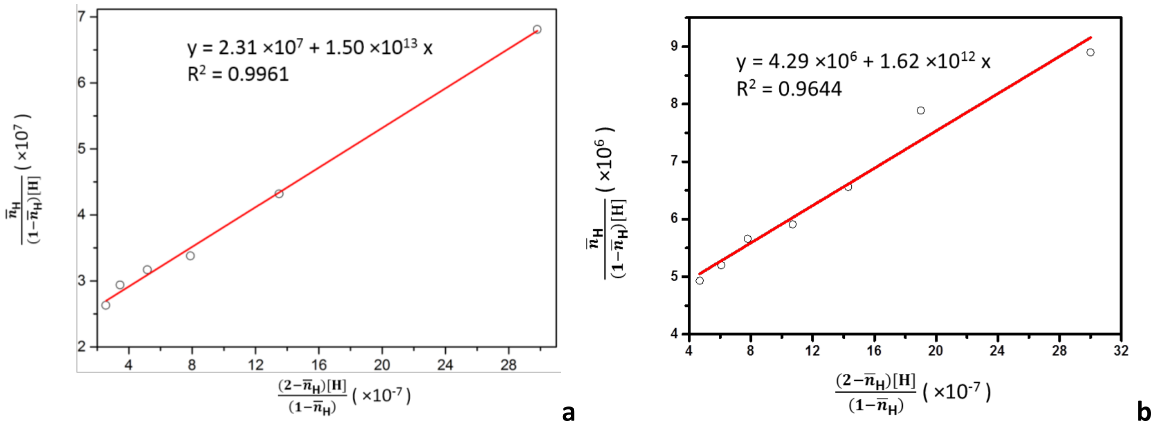

Supplementary Figure 8. Linear fitting of the data using equation (**10**) for MOC-16 (**a**) and MOC-39 (**b**) .

Selecting the suitable area of $\overline{n}_H$ (0.64 to 0.93), two values are determined for MOC-16 to be: β_1^H = 2.31×10^7, β_2^H = 1.50×10^{13} (rough data). Selecting the suitable area of $\overline{n}_H$ (0.45 to 0.81), two values are determined for MOC-39 to be: and β_1^H = 4.29×10^6, β_2^H = 1.62×10^{12}(rough data).

Supplementary Table 8. Data processing using the equation (**7**) with $\overline{n}_H$ ranging from 1.24 to 1.90 for MOC-16.

V_{KOH} /mL	pH	$\overline{n}_H$	$\frac{(3-\overline{n}_H)[H]}{2-\overline{n}_H}$	$\frac{\overline{n}_H-\beta_1^H(1-\overline{n}_H)[H]}{(2-\overline{n}_H)[H]^2}$
0.90	5.43	1.90	4.09×10^{-5}	5.74×10^{13}
0.95	5.52	1.84	2.19×10^{-5}	4.14×10^{13}
1.00	5.61	1.78	1.36×10^{-5}	3.48×10^{13}
1.05	5.68	1.72	9.55×10^{-6}	2.99×10^{13}
1.10	5.75	1.66	7.02×10^{-6}	2.67×10^{13}
1.15	5.84	1.60	5.04×10^{-6}	2.60×10^{13}

1.25	6.02	1.49	2.83×10^{-6}	2.64×10^{13}
1.35	6.17	1.36	1.73×10^{-6}	2.39×10^{13}
1.45	6.32	1.24	1.11×10^{-6}	2.23×10^{13}

Supplementary Table 9. Data processing using the equation (**7**) with $\overline{n}_H$ ranging from 1.38 to 1.80 for MOC-39.

V_{KOH}/mL	pH	$\overline{n}_H$	$\frac{(3-\overline{n}_H)[H]}{2-\overline{n}_H}$	$\frac{\overline{n}_H-\beta_1^H(1-\overline{n}_H)[H]}{(2-\overline{n}_H)[H]^2}$
0.95	5.17	1.80	4.06×10^{-5}	2.74×10^{12}
1.00	5.24	1.75	2.88×10^{-5}	2.45×10^{12}
1.05	5.31	1.69	2.07×10^{-5}	2.18×10^{12}
1.10	5.39	1.63	1.51×10^{-5}	2.06×10^{12}
1.15	5.46	1.57	1.15×10^{-5}	1.94×10^{12}
1.20	5.56	1.50	8.25×10^{-6}	1.96×10^{12}
1.25	5.63	1.44	6.52×10^{-6}	1.91×10^{12}
1.30	5.70	1.38	5.22×10^{-6}	1.87×10^{12}

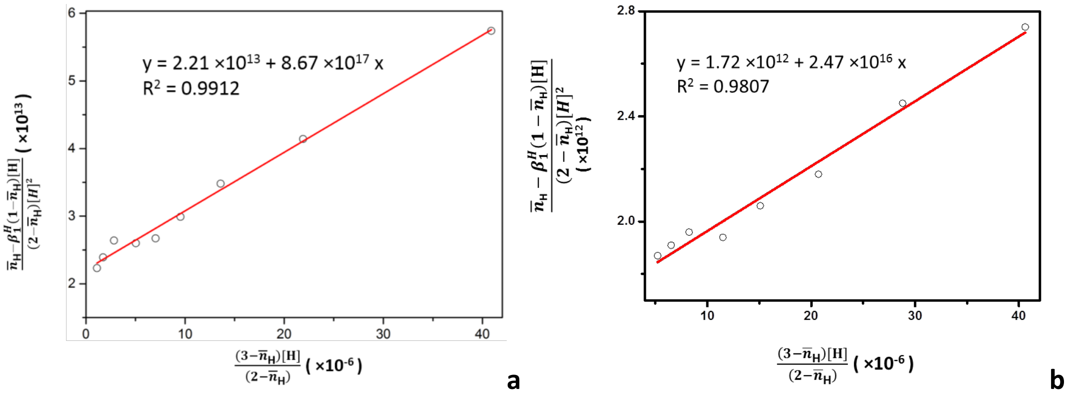

Supplementary Figure 9. Linear fitting of the data using equation (**11**) for MOC-16 (**a**) and MOC-39 (**b**).

Then, selecting the suitable area of $\overline{n}_H$ (1.24 to 1.90), the two values are determined for MOC-16 to be β_2^H = 2.21×10^{13}, β_3^H = 8.67×10^{17}, and selecting the suitable area of $\overline{n}_H$ (1.38 to 1.80), the two values are determined for MOC-39 to be β_2^H = 2.21×10^{13}, β_3^H = 8.67×10^{17}.

According to equation (**12**), the three pK_a values are determined for MOC-16 and MOC-39 to be:

$$\mathbf{p}K_{a1av}^{Pd} = \mathbf{4.59\ (0.02),\ p}K_{a2av}^{Pd} = \mathbf{5.98\ (0.02),\ p}K_{a3av}^{Pd} = \mathbf{7.36\ (0.03)}$$

$$\mathbf{p}K_{a1av}^{Pt} = \mathbf{4.16\ (0.03),\ p}K_{a2av}^{Pt} = \mathbf{5.60\ (0.03),\ p}K_{a3av}^{Pt} = \mathbf{6.63\ (0.04)}$$

4.3 Theoretical calculations

To reveal the cage's ionization activity in water, considering that the dimension of the system is rather large, the semi-empirical quantum approach was selected to calculate its Gibbs free energy. All the calculations were carried on MOPAC2016 (*5*). We used PM6 as diffuse function treat organic system while the D3H4 correction (*6*) was implemented to describe the hydrogen bonding and the dispersion contribution more accurately. In details, the correction to the dispersion uses Grimme's D3 method (*7*) while the hydrogen bonding is described by the "H4" hydrogen-bond function developed by Řezáč and Hobza (*8*). This approach was already successfully applied to diverse systems including drug (*9*), graphene materials (*10*) and macromolecules (*11*).

The Gibbs free energy variation of each consecutive deprotonation step corresponding to a release of 1 –H from the –NH function of the imidazole group of the cage was further calculated following the equation mentioned below. The total number of –H atoms potentially involved in the deprotonation process is 24 indeed m can vary from 0 to 24.

$$Cage + 24H_2O \rightarrow [Cage]^{m-} + [mH_3O^+ + (24-m)H_2O] \quad m \in [1,24], m \in N^*$$
$$\Delta G_r = G[mH_3O^+ + (24-m)H_2O] + G\,[Cage]^{m-} - G(Cage) - G(24H_2O)$$

The model for the cage was obtained from single-crystal X-ray data while the cage^{m-} corresponds to the ionized structure resulting from the transfer of m protons to adsorbed water molecules. The protons for release and the water to host the protons were selected randomly at each deprotonation step. Besides the cage system, we further built a cubic cell with a length of 20 Å, containing 24 H_2O molecules, as another reactant present in the equation mentioned above (Fig. S10). Moreover, the same cells containing (24-m) H_2O molecules and m H_3O^+ molecules as a product were further considered. All the corresponding systems were geometry optimized under solvent environment using the COSMO solvation model (*12*). According to the equation, the numbers of hydrogen atoms on the cage potentially involved in the deprotonation process can be evaluated thermodynamically with the calculation of ΔG for each dehydrogenation step.

Fig. S11 that reports the evolution of ΔG_r for each deprotonation process evidences that it is energetically favorable to release 1 to 4 hydrogen atoms from the cage. The next step was to validate the most preferential location of the protons once released from the cage. To that purpose, a proton was thus initially placed on each of the 24 available H_2O molecules and the system was geometry optimized for each corresponding configuration and the Gibbs free energy was evaluated to reveal the lowest energetic configurations. For the lowest configurations found for both inside and outside cages, we further calculated the ΔG_r corresponding to the release of this proton. We found that when proton combines to a H_2O molecule outside the cage, ΔG_{min} = -42.52kJ/mol, while ΔG_{min} = -

29.17kJ/mol for inside the cage (Fig. S12). This emphasizes that it is more energetically favorable to make the proton transfer with one molecule present outside the cage that is thus expected to induce a variation of the pH making the environment more acid. This is validated by the experimental catalytic data.

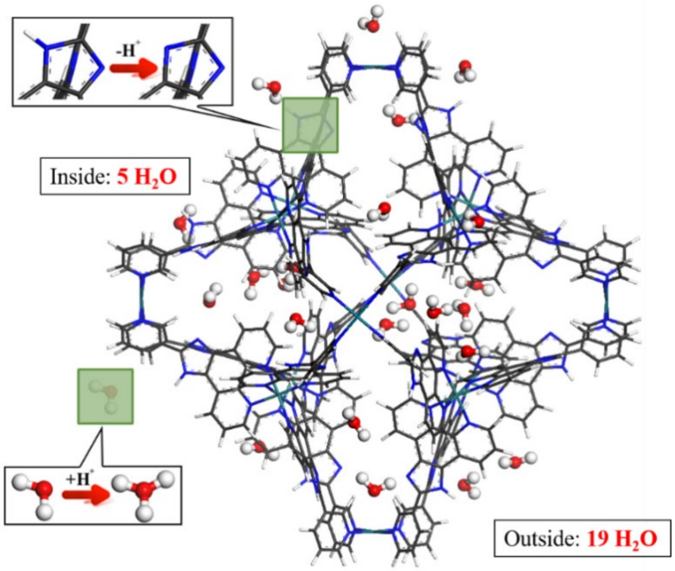

Supplementary Figure 10. The cage model used for the calculation of the free Gibbs energy variation corresponding to the deprotonation process.

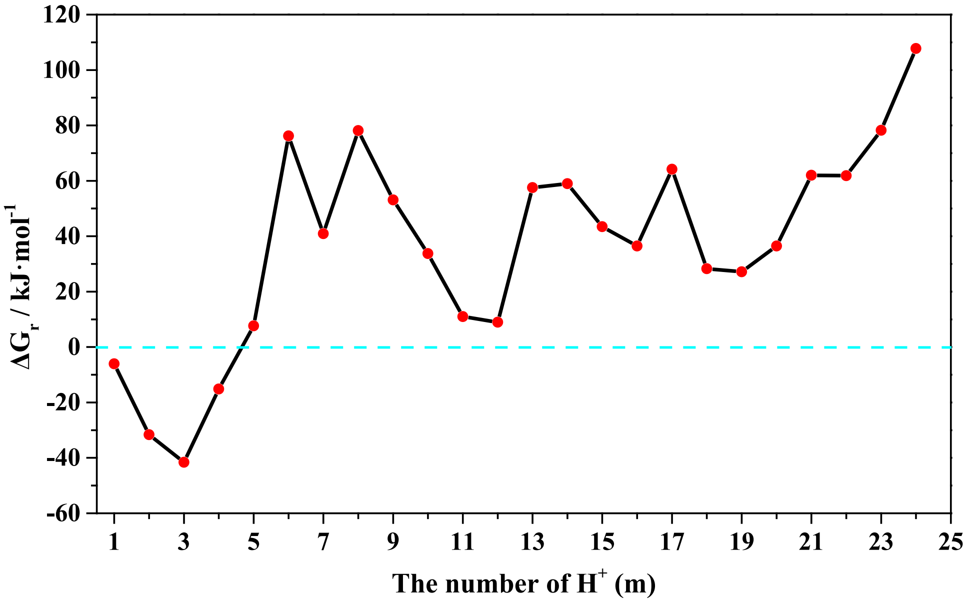

Supplementary Figure 11. The free Gibbs energy profile of the sequential deprotonation of the cage calculated using the equation mentioned above.

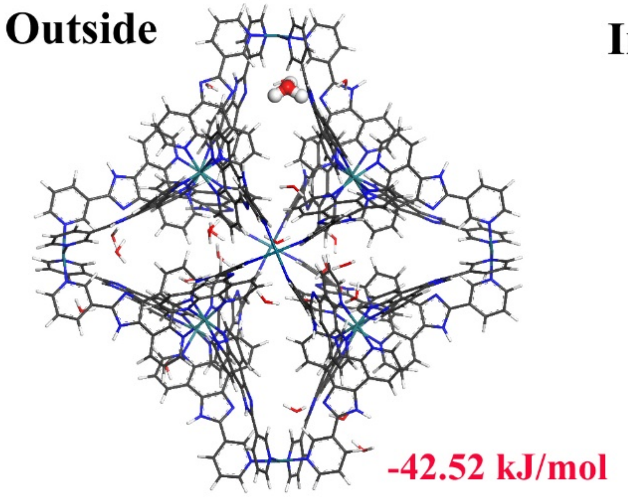

Supplementary Figure 12. The Gibbs free energy variation calculated from the preferential configuration of protons localized on H_2O present either outside or inside the cages.

4.4 Acid stability of MOC-16/39

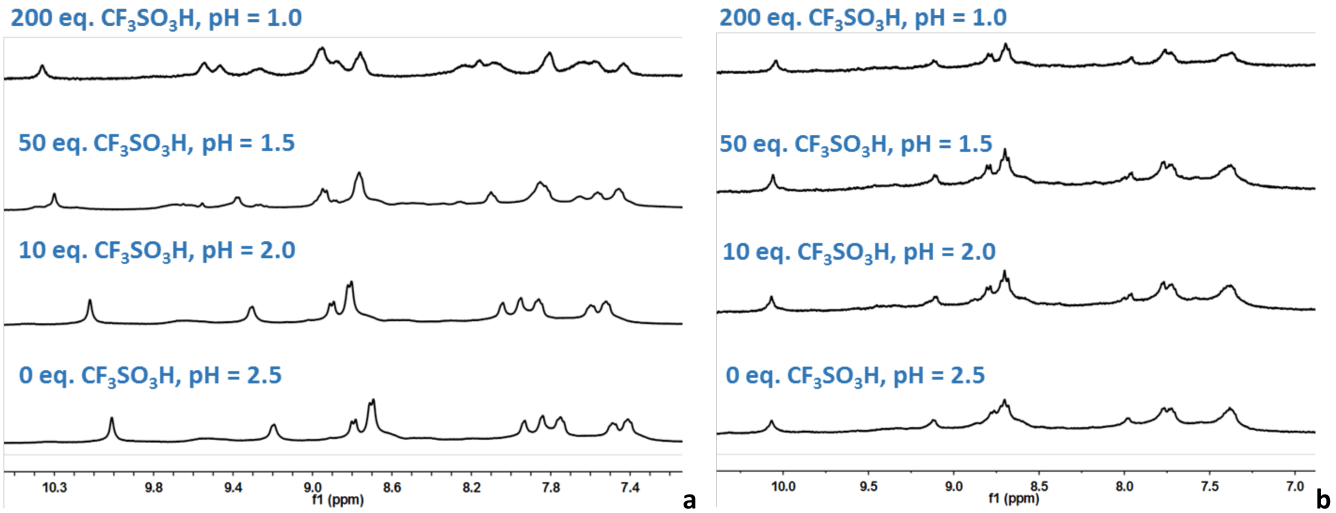

Supplementary Figure 13. Chemical stability monitored by ^{1}H NMR spectra. (**a**) MOC-16 with CF_3SO_3H in DMSO-d_6-D_2O (v: v = 1: 10). (**b**) MOC-39 with CF_3SO_3H in DMSO-d_6-D_2O (v: v = 1: 10).

5. Detailed study of H/D-exchange and Knoevenagel-condensation

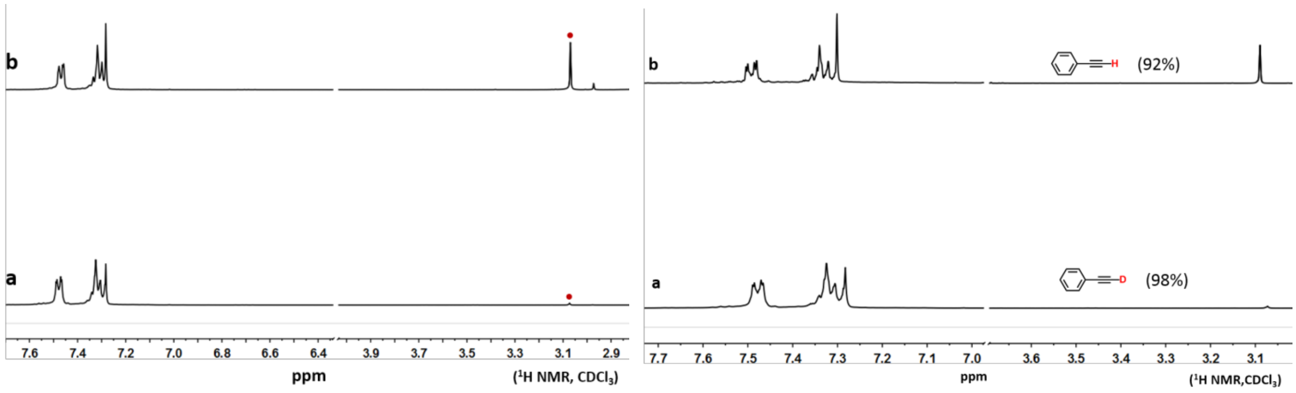

Supplementary Figure 14. Left, comparison of deuteration of phenylacetylene in the presence of MOC-16 for 7 h at r.t. under different media: (a) In DMSO-d_6/D_2O (v/v = 1/10). (b) DMSO-d_6/H_2O (v/v = 1/10). Right, reversible H/D exchange process for phenylacetylene in the presence of MOC-16. (a) 98 % deuterated phenylacetylene showing residual terminal H of ethynyl group. (b) The terminal deuterium was exchanged back to proton in the presence of MOC-16 within a mixture of DMSO-d_6-H_2O (0.3 mL/3.0 mL, v/v) for 7 h at r.t.

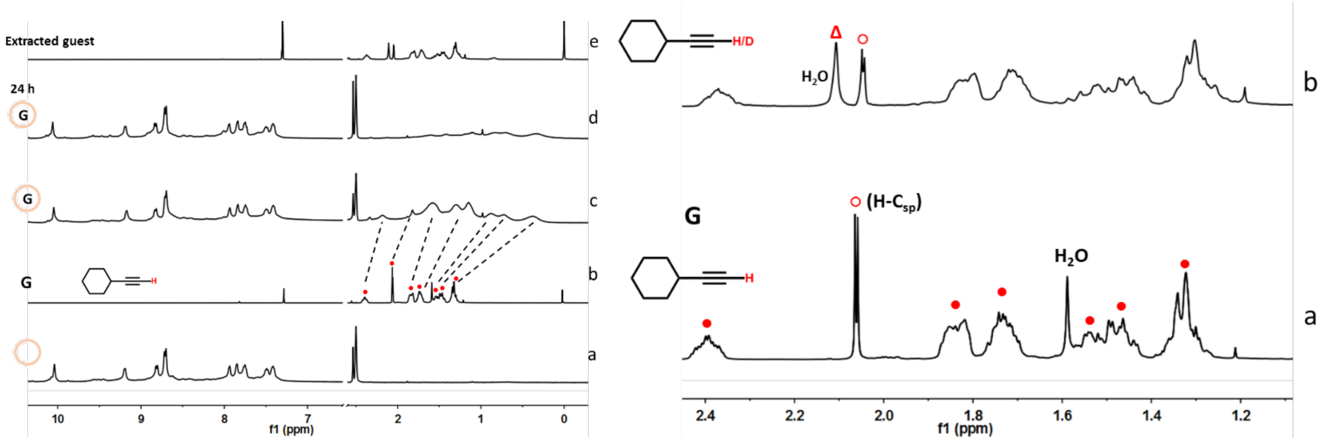

Supplementary Figure 15. Deuteration of ethynylcyclohexane (denoted by G) through encapsulation inside MOC-16 in DMSO-d_6:D_2O (1: 10 v/v, pD = 2.5) at r.t. Left: (a) MOC-16. (b) Cyclohexylacetylene ($CDCl_3$). (c) The host-guest solution at the beginning. (d) The solution kept for 24 h. (e) The extracted guest from d ($CDCl_3$). Right: (a) Cyclohexylacetylene before adding into MOC-16 solution ($CDCl_3$). (b) After deuteration in MOC-16 solution for 24 h, showing 40% H/D exchange.

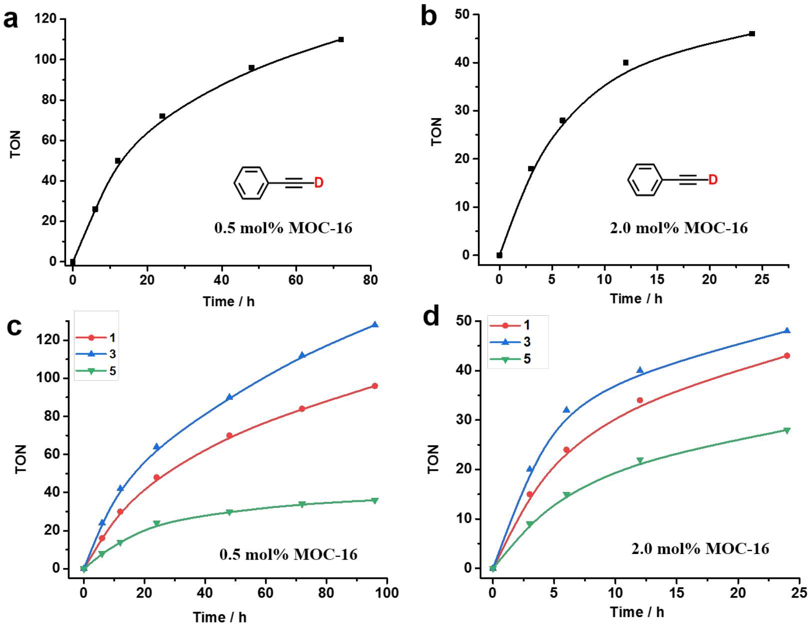

Supplementary Figure 16. TON vs time plots for representative H/D-exchange and Knoevenagel condensation reactions in the presence of 0.5 and 2 mol% loading of MOC-16 as catalyst. (a-b) H/D exchange for phenylacetylene; (b-c) Knoevenagel condensation for benzaldehyde (**1**), 4-fluorobenzaldehyde (**2**) and 6-methoxy-2-naphthaldehyde (**3**).

6. Detailed study of acid/base cascade catalysis

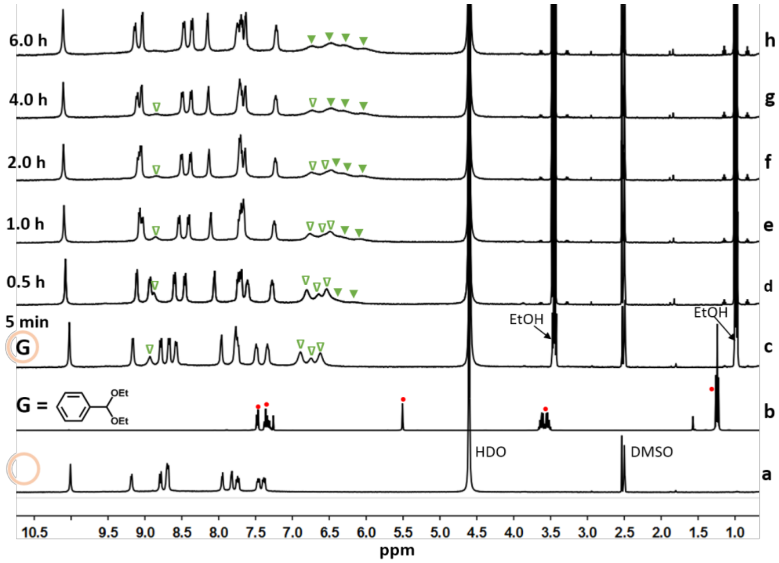

Supplementary Figure 17. In situ ^{1}H NMR spectra monitoring of the one-pot cascade reaction of benzaldehyde diethyl acetal and malonontrile in the presence of MOC-16 (400 MHz, 300K). (**a**) MOC-16 (DMSO-d_6:D_2O = 1:10, v/v), pD = 2.5. (**b**) Benzaldehyde diethyl acetal ($CDCl_3$). (**c**) Host-guest solution between MOC-16 (0.004 mmol) and benzaldehyde diethyl acetal (0.024 mmol) kept for 5 min, and 0.5 h (**d**), 1.0 h (**e**), 2.0 h (**f**), 4.0 h (**g**), 6.0 h (**h**) at r.t. The hollow triangles represent the hydrolysis product as aldehyde, and the green triangles represent the final condensation product.

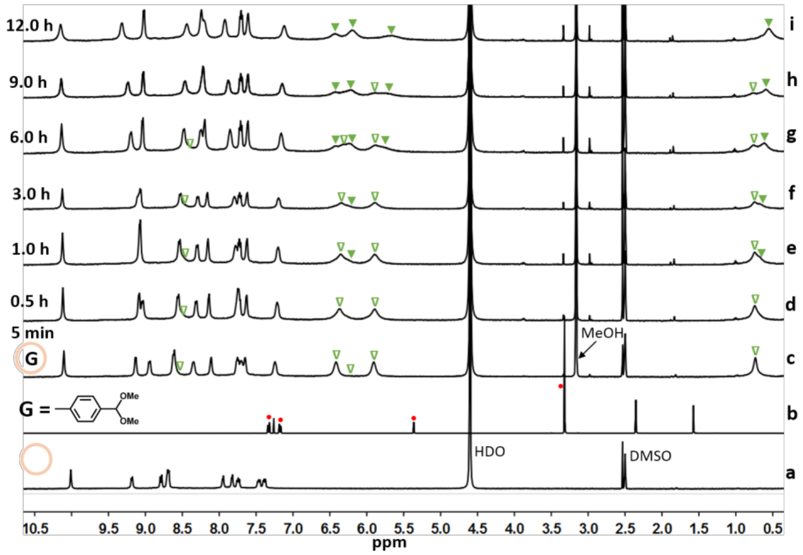

Supplementary Figure 18. In situ ^{1}H NMR spectra monitoring of the one-pot cascade reaction of p-methylbenzaldehyde dimethyl acetal and malonontrile in the presence of MOC-16 (400 MHz, 300K). (**a**) MOC-16 (DMSO-d_6:D_2O = 1:10, v/v), pD = 2.5. (**b**) p-Methylbenzaldehyde dimethyl acetal ($CDCl_3$). (**c**) Host-guest solution between MOC-16 (0.004 mmol) and p-methylbenzaldehyde dimethyl acetal (0.024 mmol) kept for 5 min, and 0.5 h (**d**), 1.0 h (**e**), 3.0 h (**f**), 6.0 h (**g**), 9.0 h (**h**), 12.0 (**i**) at r.t. The hollow triangles represent the hydrolysis product as aldehyde, and the green triangles represent the final condensation product.

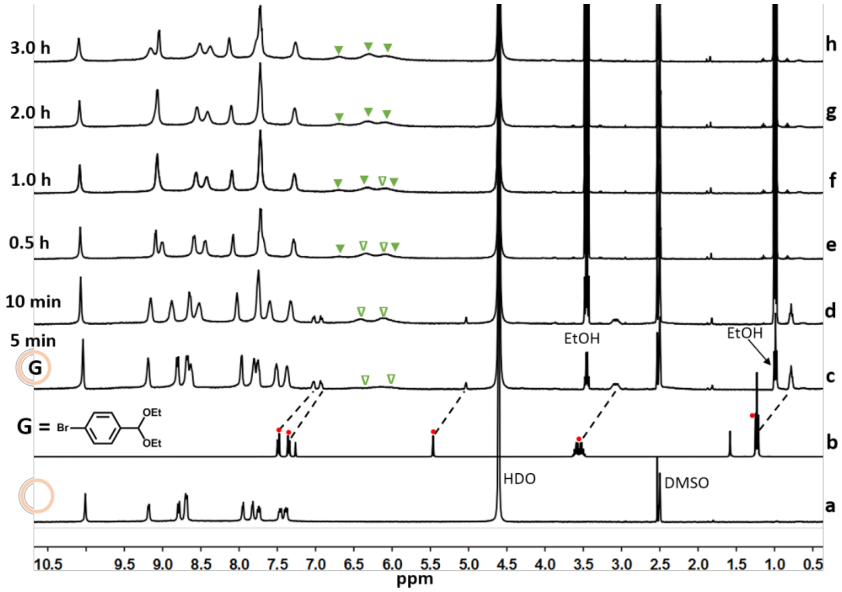

Supplementary Figure 19. In situ ^{1}H NMR spectra monitoring of the one-pot cascade reaction of 4-bromobenzaldehyde diethyl acetal and malonontrile in the presence of MOC-16 (400 MHz, 300K). (**a**) MOC-16 (DMSO-d_6:D_2O = 1:10, v/v), pD = 2.5. (**b**) 4-Bromobenzaldehyde diethyl acetal ($CDCl_3$). (**c**) Host-guest solution between MOC-16 (0.004 mmol) and 4-bromobenzaldehyde diethyl acetal (0.024 mmol) kept for 5 min, and 10 min (**d**), 0.5 h (**e**), 1.0 h (**f**), 2.0 h (**g**), 3.0 h (**h**) at r.t The hollow triangles represent the hydrolysis product as aldehyde, and the green triangles represent the final condensation product.

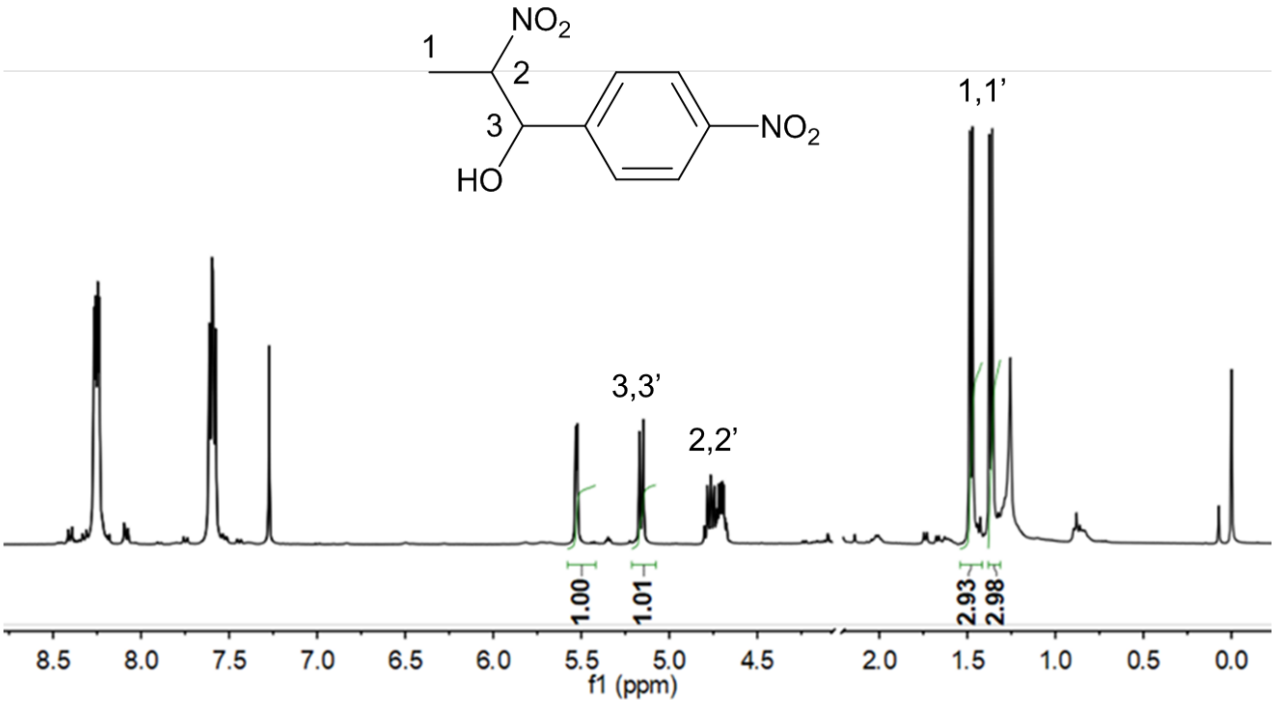

Supplementary Figure 20. ^{1}H NMR spectrum of compound **14**. (400 MHz, $CDCl_3$)

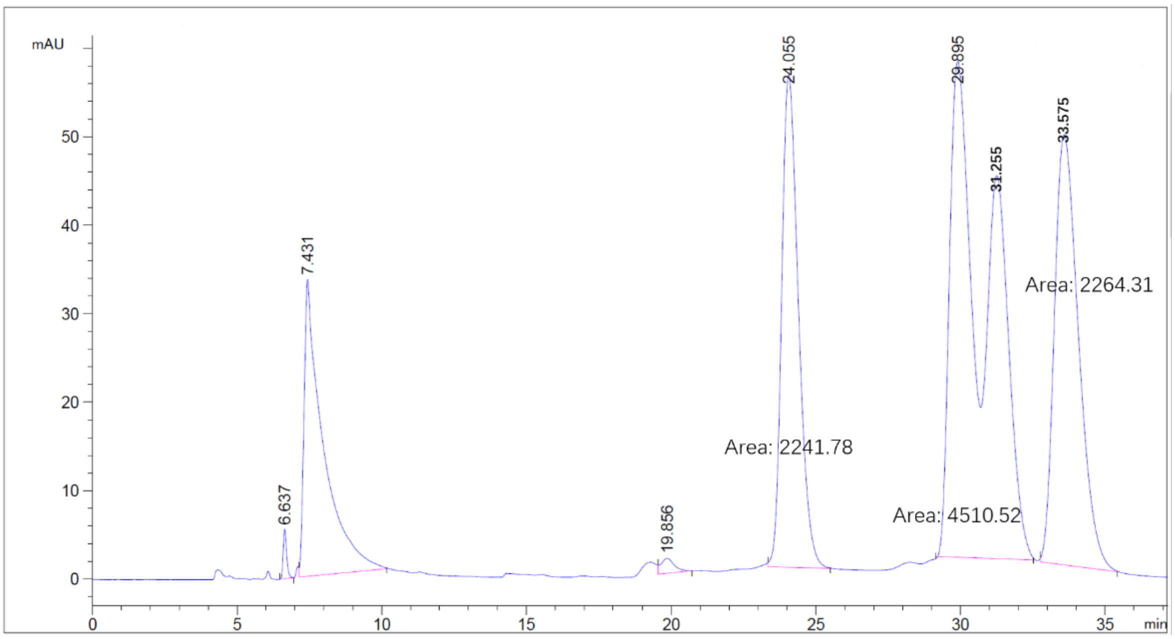

Supplementary Figure 21. The HPLC of compound **14** catalyzed by *Δ*-MOC-16. (Daicel_OD-H column, 1.0 mL/min, 95% n-hexane, 5% isopropanol, RT)

Supplementary Table 10. Yields (%) of cascade reactions of varied acetal derivatives at different pH values.

Entry	pH = 1.5		pH = 2.5		pH = 4.0		pH = 6.0		pH = 8.0	
	**With MOC-16**	**Without MOC-16**	**With MOC-16**	**Without MOC-16**	**With MOC-16**	**Without MOC-16**	**With MOC-16**	**Without MOC-16**	**With MOC-16**	**Without MOC-16**
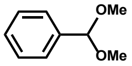	89	0	95	3	95	23	19	5	0	0
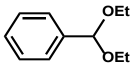	87	0	90	3	92	22	33	6	0	0
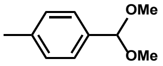	90	0	93	0	95	14	33	9	0	0
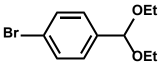	96	0	99	0	94	11	2	0	0	0

Reaction conditions: MOC-16 (0.004 mmol), acetal (0.024 mmol), malonontrile (0.024 mmol), DMSO (0.3 mL), H_2O (3.0 mL), r.t., 12 h. The yields were determined by ^{1}H NMR using mesitylene as internal standard.

7. Detailed study of A^3-coupling catalysis

Supplementary Table 11. A^3-coupling reactions of benzaldehyde, aniline and phenylacetylene under different conditions

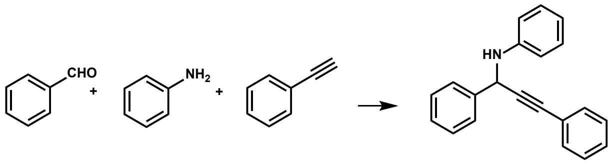

Entry	Catalyst	Solvent	Yield[g] / %
1[a]	CuOTf	DMSO-H_2O (1/4, v/v)	12
2[b]	CuOTf + RuL_3	DMSO-H_2O (1/4, v/v)	8
3[c]	MOC-16@CuOTf	DMSO-H_2O (1/4, v/v)	80
4[d]	CuOTf	Toluene	32
5[e]	CuOTf + NEt_3	Toluene	85

Reaction conditions: benzaldehyde (0.200 mmol), aniline (0.240 mmol), phenylacetylene (0.300 mmol), solvent (0.5 mL), 60 ℃ for 6 h, N_2 atmosphere. [a] CuOTf (0.020 mmol), [b] CuOTf (0.020 mmol), RuL_3 (0.008 mmol), [c] CuOTf (0.020 mmol) in MOC-16 (0.001 mmol) solution. [d] CuOTf (0.020 mmol). [e] CuOTf (0.020 mmol), NEt_3 (0.300 mmol). [g] ^{1}H NMR yield.

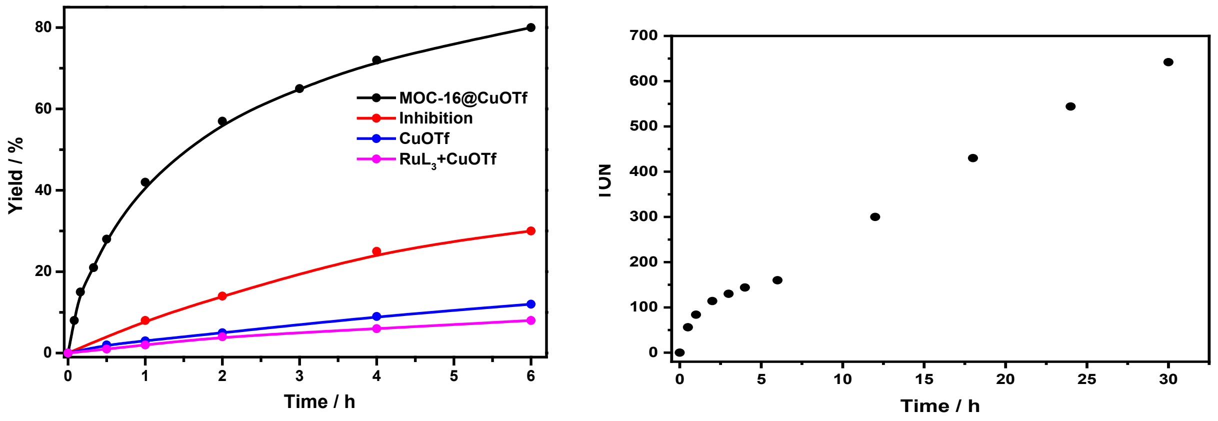

a b

Supplementary Figure 22. (**a**) Comparison of the A^3-coupling conversions *vs.* time under different conditions in aqueous solution (pH = 2.5). 1,1'-diacetyl ferrocene was used as competeing guest. (**b**) Accumulative TON of A^3-coupling of PhCHO, $PhNH_2$ and phenylacetylene for 5 successive recycling reactions with addition of aliquots substrates (200 eq.) at 6 h intervals after extraction of the product.

$$\frac{1}{2}\left[\frac{1}{(a-x)^2} - \frac{1}{a^2}\right] = k_3 t$$

a : initial concentration of substrate, a-x : substrate concentration at t, t : reaction time

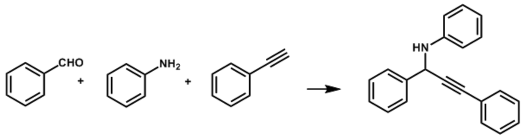

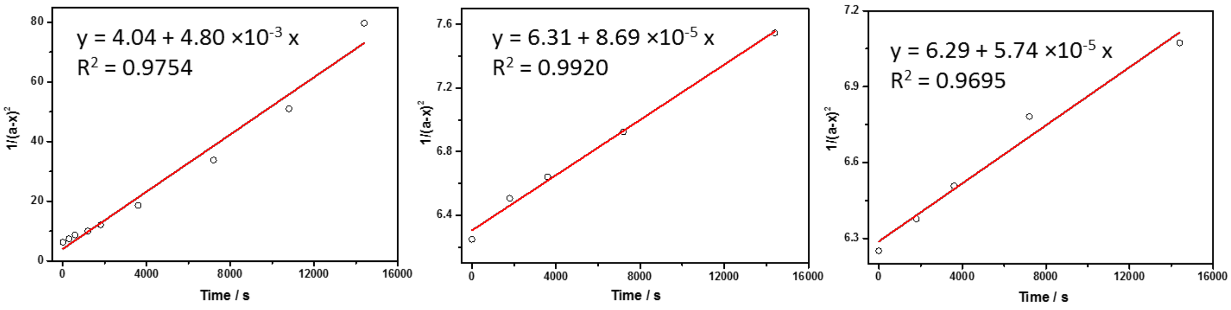

Catalyst: MOC-16@CuOTf
$k_3 = 4.80 \times 10^{-3}\ M^{-2}{\bullet}s^{-1}$

Catalyst: CuOTf
$k_3 = 8.69 \times 10^{-5}\ M^{-2}{\bullet}s^{-1}$

Catalyst: RuL_3 + CuOTf
$k_3 = 5.74 \times 10^{-5}\ M^{-2}{\bullet}s^{-1}$

$k_{3(\text{MOC-16@CuOTf})} / k_{3(\text{CuOTf})} \approx 55$ $k_{3(\text{MOC-16@CuOTf})} / k_{3(\text{RuL3 + CuOTf})} \approx 84$

Supplementary Figure 23. Kinetics of A^3-coupling three-order reactions under different conditions in aqueous solution (pH = 2.5)

Supplementary Table 12. A^3-coupling reactions of 4-methylbenzaldehyde, aniline and phenylacetylene under different conditions

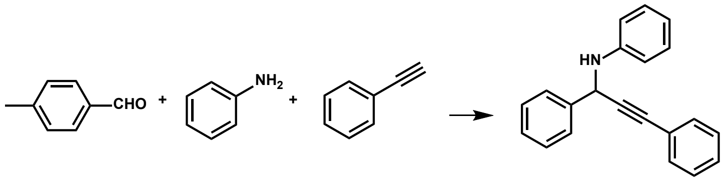

Entry	Catalyst	Solvent	Yield[g] / %
1[a]	CuOTf	DMSO-H_2O (1/4, v/v)	9
2[b]	CuOTf + RuL_3	DMSO-H_2O (1/4, v/v)	6
3[c]	MOC-16@CuOTf	DMSO-H_2O (1/4, v/v)	75
4[d]	CuOTf	Toluene	15
5[e]	CuOTf + NEt_3	Toluene	69

Reaction conditions: 4-methylbenzaldehyde (0.200 mmol), aniline (0.240 mmol), phenylacetylene (0.300 mmol), solvent (0.5 mL), 60 ℃ for 6 h, N_2 atmosphere. [a] CuOTf (0.020 mmol), [b] CuOTf (0.020 mmol), RuL_3 (0.008 mmol), [c] CuOTf (0.020 mmol) in MOC-16 (0.001 mmol) solution. [d] CuOTf (0.020 mmol). [e] CuOTf (0.020 mmol), NEt_3 (0.300 mmol). [g] ^1H NMR yield.

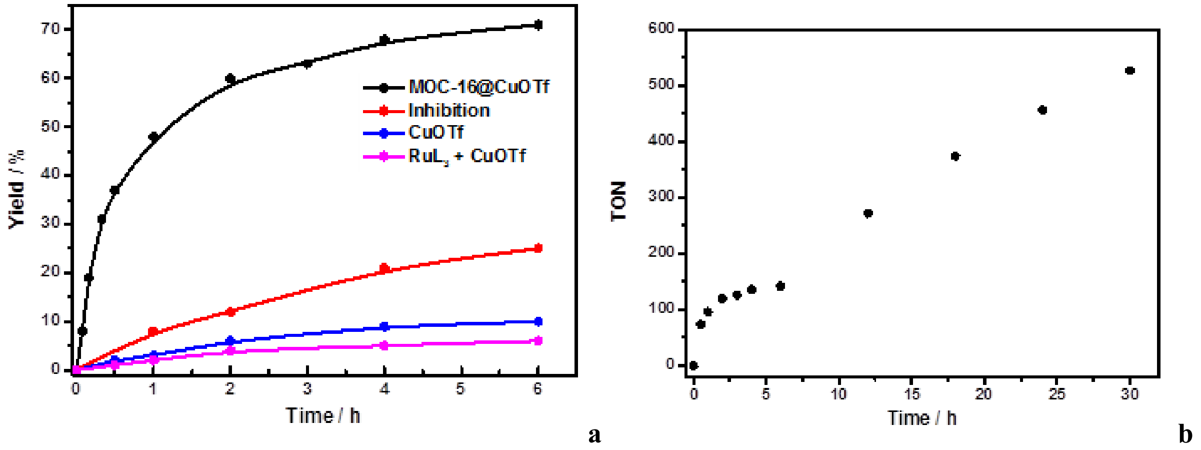

Supplementary Figure 24. (**a**) Comparison of the A^3-coupling conversions *vs.* time under different conditions in aqueous solution (pH = 2.5). 1,1'-diacetyl ferrocene was used as competeing guest. (**b**) Accumulative TON of A^3-coupling of MePhCHO, $PhNH_2$ and phenylacetylene for 5 successive recycling reactions with addition of aliquots substrates (200 eq.) at 6 h intervals after extraction of the product.

$$\frac{1}{2}\left[\frac{1}{(a-x)^2} - \frac{1}{a^2}\right] = k_3 t$$

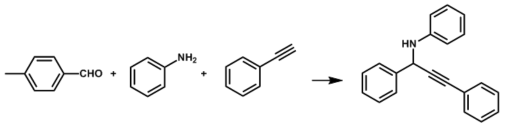

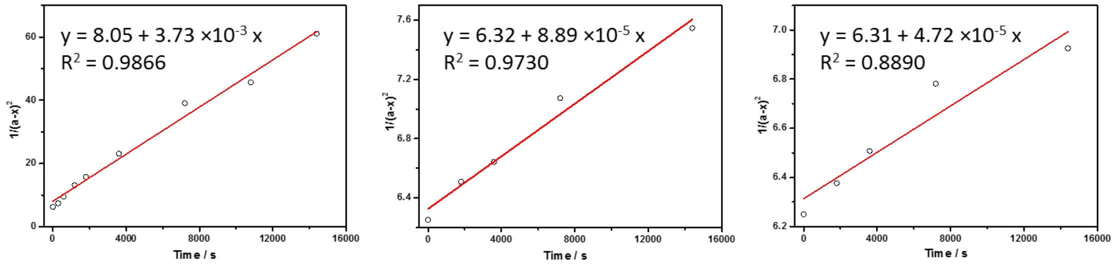

Catalyst: MOC-16@CuOTf
k_3 = 3.73 ×10^{-3} M^{-2}•s^{-1}

Catalyst: CuOTf
k_3 = 8.89 ×10^{-5} M^{-2}•s^{-1}

Catalyst: RuL_3 + CuOTf
k_3 = 4.72 ×10^{-5} M^{-2}•s^{-1}

$k_{3(\text{MOC-16@CuOTf})} / k_{3(\text{CuOTf})} \approx 42$ $\quad$ $k_{3(\text{MOC-16@CuOTf})} / k_{3(\text{RuL3 + CuOTf})} \approx 79$

Supplementary Figure 25. Kinetics of A^3-coupling three-order reactions under different conditions in aqueous solution (pH = 2.5).

Supplementary Table 13. A^3-coupling reactions of benzaldehyde, aniline and phenylacetylene under different conditions

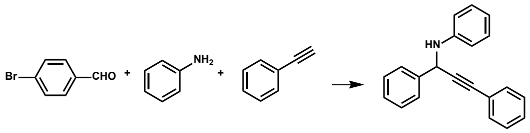

Entry	Catalyst	Solvent	Yield[g] / %
1[a]	CuOTf	DMSO-H_2O (1/4, v/v)	11
2[b]	CuOTf + RuL_3	DMSO-H_2O (1/4, v/v)	7
3[c]	MOC-16@CuOTf	DMSO-H_2O (1/4, v/v)	90
4[d]	CuOTf	Toluene	20
5[e]	CuOTf + NEt_3	Toluene	95

Reaction conditions: 4-bromobenzaldehyde (0.200 mmol), aniline (0.240 mmol), phenylacetylene (0.300 mmol), solvent (0.5 mL), 60 ℃ for 6 h, N_2 atmosphere. [a] CuOTf (0.020 mmol), [b] CuOTf (0.020 mmol), RuL3 (0.008 mmol), [c] CuOTf (0.020 mmol) in MOC-16 (0.001 mmol) solution. [d] CuOTf (0.020 mmol). [e] CuOTf (0.020 mmol), NEt_3 (0.300 mmol). [g] ^1H NMR yield.

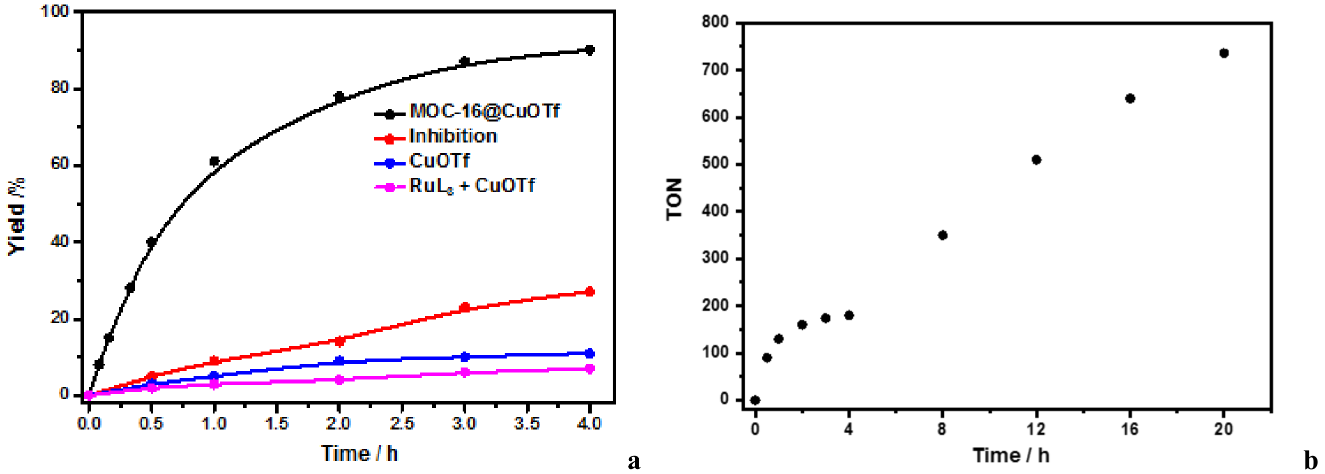

Supplementary Figure 26. (**a**) Comparison of the A^3-coupling conversions *vs.* time under different conditions in aqueous solution(pH = 2.5). 1,1'-diacetyl ferrocene was used as competeing guest. (**b**) Accumulative TON of A^3-coupling of 4-BrPhCHO, $PhNH_2$ and phenylacetylene for 5 successive recycling reactions with addition of aliquots substrates (200 eq.) at 6 h intervals after extraction of the product.

$$\frac{1}{2}\left[\frac{1}{(a-x)^2} - \frac{1}{a^2}\right] = k_3 t$$

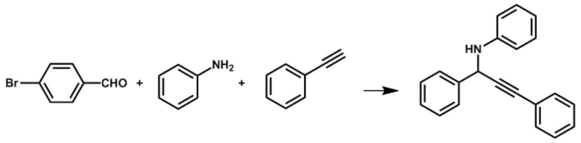

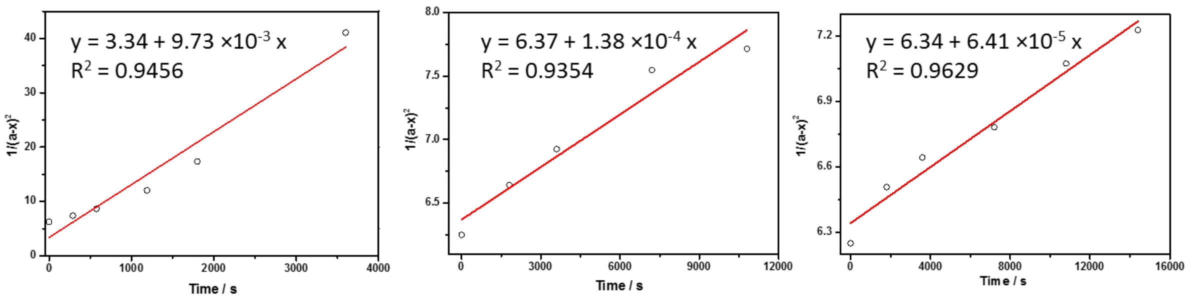

Catalyst: MOC-16@CuOTf
$k_3 = 9.73 \times 10^{-3}\ M^{-2} \cdot s^{-1}$

Catalyst: CuOTf
$k_3 = 1.38 \times 10^{-4}\ M^{-2} \cdot s^{-1}$

Catalyst: RuL_3 + CuOTf
$k_3 = 6.41 \times 10^{-5}\ M^{-2} \cdot s^{-1}$

$k_{3(\text{MOC-16@CuOTf})} / k_{3(\text{CuOTf})} \approx 71$

$k_{3(\text{MOC-16@CuOTf})} / k_{3(\text{RuL3 + CuOTf})} \approx 152$

Supplementary Figure 27. Kinetics of A^3-coupling three-order reactions under different conditions in aqueous solution (pH = 2.5).

Supplementary Table 14. Representative catalytic reactions with MOC-39 under different acidic extrinsic media.

Entry	Reactions	Yield/% (pH = 2.5)	Yield/% (pH = 1.0)[d]
1[a]	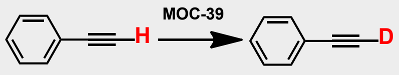	90	65
2[b]	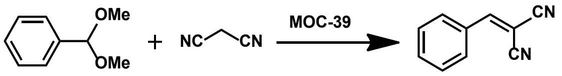	93	56
3[b]	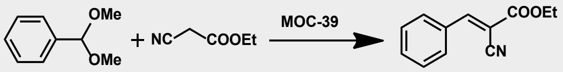	91	53
4[b]	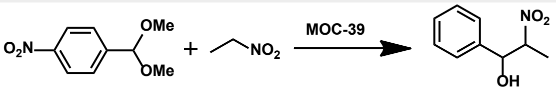	94	48
5[c]	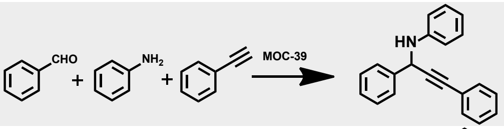	83	57
6[c]	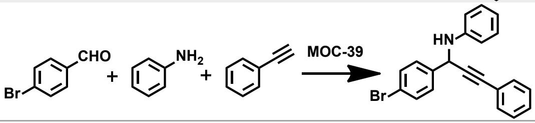	90	62

[a] 17 mol% MOC-39, DMSO/D_2O = 1/10 (v/v), r.t. for 7 h. [b] 17 mol% MOC-139, DMSO/H_2O = 1/10 (v/v), r.t. for 12 h. [c] 0.5 mol% MOC-39, 10 mol% CuOTf, DMSO/H_2O = 1/4 (v/v), N_2, 60 °C for 6 h. [d] The pH values were adjusted by adding CF_3SO_3H.

8. References:

1. Li K, Zhang LY, Yan C *et al.* Stepwise assembly of $Pd_6(RuL_3)_8$ nanoscale rhombododecahedral metal-organic cages via metalloligand strategy for guest trapping and protection. *J Am Chem Soc* 2014; **136**: 4456-59.
2. Sheldrick GM. A short history of SHELX. *Acta Crystallogr A* 2008; **64**, 112-22.
3. Spek AL. PLATON SQUEEZE: a tool for the calculation of the disordered solvent contribution to the calculated structure factors. *Acta Crystallogr C* 2015; **71**, 9-18.
4. Edward RB, Elbert AW. A mathematical analysis of the bjerrum function for the stepwise equilibrium model. *J Math Chem* 1987; **1**, 235-47.
5. Stewart JJP. MOPAC2016, version: 16.175 W. Stewart Computational Chemistry. *Colorado Springs, CO, USA* http://OpenMOPAC. net.
6. Rezac J, Hobza P. Advanced corrections of hydrogen bonding and dispersion for semiempirical quantum mechanical methods. *J Chem Theory Comput* 2012; **8**, 141-51.
7. Grimme S, Antony J, Ehrlich S *et al.* A consistent and accurate ab initio parametrization of density functional dispersion correction (DFT-D) for the 94 elements H-Pu. *J Chem Phys* 2010; **132**, 1-13.
8. Vorlová B, Nachtigallová D, Jirásková-Vaníčková J *et al.* Malonate-based inhibitors of mammalian serine racemase: Kinetic characterization and structure-based computational study. *Eur J Med Chem* 2015; **89**, 189-97.
9. Lepšík M, Rezác J, Kolár M *et al.*, The Semiempirical quantum mechanical scoring function for In silico drug design. *Chempluschem* 2013; **78**, 921-31.
10. Sedykh AE, Gordeev EG, Pentsak EO *et al.* Shielding the chemical reactivity using graphene layers for controlling the surface properties of carbon materials. *Phys Chem Chem Phys* 2016; **18**, 4608-16.
11. Jin XY, Wang F, Cong H *et al.* Host-guest interactions between hemicucurbiturils and a hydroxyl-substituted Schiff base. *J Incl Phenom Macro* 2016; **86**, 249-54.

12. Klamt A, Schüürmann G. COSMO: a new approach to dielectric screening in solvents with explicit expressions for the screening energy and its gradient. *J Chem Soc, Perkin Trans.* 1993; ***2*0**, 799-805.
